# Novel Biomarker Proteins in Chronic Lymphocytic Leukemia: Impact on Diagnosis, Prognosis and Treatment

**DOI:** 10.1371/journal.pone.0148500

**Published:** 2016-04-14

**Authors:** Lee Admoni-Elisha, Itay Nakdimon, Anna Shteinfer, Tal Prezma, Tasleem Arif, Nir Arbel, Anna Melkov, Ori Zelichov, Itai Levi, Varda Shoshan-Barmatz

**Affiliations:** 1 Department of Life Sciences and the National Institute for Biotechnology in the Negev, Ben-Gurion University of the Negev, Beer Sheva, Israel; 2 Department of Hematology, Soroka University Medical Center and the Faculty of Health Sciences, Ben-Gurion University of the Negev, Beer Sheva, Israel; European Institute of Oncology, ITALY

## Abstract

In many cancers, cells undergo re-programming of metabolism, cell survival and anti-apoptotic defense strategies, with the proteins mediating this reprogramming representing potential biomarkers. Here, we searched for novel biomarker proteins in chronic lymphocytic leukemia (CLL) that can impact diagnosis, treatment and prognosis by comparing the protein expression profiles of peripheral blood mononuclear cells from CLL patients and healthy donors using specific antibodies, mass spectrometry and binary logistic regression analyses and other bioinformatics tools. Mass spectrometry (LC-HR-MS/MS) analysis identified 1,360 proteins whose expression levels were modified in CLL-derived lymphocytes. Some of these proteins were previously connected to different cancer types, including CLL, while four other highly expressed proteins were not previously reported to be associated with cancer, and here, for the first time, DDX46 and AK3 are linked to CLL. Down-regulation expression of two of these proteins resulted in cell growth inhibition. High DDX46 expression levels were associated with shorter survival of CLL patients and thus can serve as a prognosis marker. The proteins with modified expression include proteins involved in RNA splicing and translation and particularly mitochondrial proteins involved in apoptosis and metabolism. Thus, we focused on several metabolism- and apoptosis-modulating proteins, particularly on the voltage-dependent anion channel 1 (VDAC1), regulating both metabolism and apoptosis. Expression levels of Bcl-2, VDAC1, MAVS, AIF and SMAC/Diablo were markedly increased in CLL-derived lymphocytes. VDAC1 levels were highly correlated with the amount of CLL-cancerous CD19+/CD5+ cells and with the levels of all other apoptosis-modulating proteins tested. Binary logistic regression analysis demonstrated the ability to predict probability of disease with over 90% accuracy. Finally, based on the changes in the levels of several proteins in CLL patients, as revealed from LC-HR-MS/MS, we could distinguish between patients in a stable disease state and those who would be later transferred to anti-cancer treatments. The over-expressed proteins can thus serve as potential biomarkers for early diagnosis, prognosis, new targets for CLL therapy, and treatment guidance of CLL, forming the basis for personalized therapy.

## Introduction

Cancer biomarkers are molecular indicators of a biological status, often produced by the tumor itself or the host system in response to the tumor, and can be used for early detection, diagnosis, prognosis, and prediction of response to treatment and cancer recurrence [1]. While deep sequencing and other genetic tools are widely accepted as means to detect and analyze such cancer biomarkers [2], many cancer-associated changes are not mutation-related but rather appear as changes in the expression level or post-translational modification of marker proteins.

Due to the complexity and heterogeneity of most solid tumors, even at a single cancer site, it is now well accepted that a single biomarker is not sufficient for disease diagnosis, progression or treatment efficacy. Proteins, as the actual functional molecules in the cell that can be identified at the expression level and in terms of post-translational modification (i.e. glycosylation, acetylation, phosphorylation, etc.) are often better suited for use as biomarkers [1,3].

The ‘hallmarks of cancer’, as defined by Hanahan and Weinberg [4], comprise a set of cellular traits thought to be necessary for tumorigenesis that include resisting cell death and reprogramming energy metabolism. Such alterations occur in CLL, a cancer characterized by an accumulation of CD19+/CD5+ B lymphocytes [5]. Evasion of apoptosis is often promoted by dysregulation of the expression of pro- and anti-apoptotic Bcl-2 family proteins [6]. Indeed, the anti-apoptotic proteins Bcl-2 and Bcl-xL are expressed at high levels in many types of cancer, including CLL [7], and were found to govern mitochondrial apoptotic responses [8]. Levels of Mcl-1, another member of the Bcl-2 family, have been correlated with more advanced forms of CLL and resistance to both chemotherapy and Bcl-2 inhibitors [9]. In addition, in a number of cancers, including advanced CLL [10,11], reduced levels of the pro-apoptotic proteins Bax or Bak contribute to chemoresistance.

In many cancers, cells undergo metabolic re-programing and rely on glycolysis as the main energy-generating pathway, even in the presence of oxygen (the ‘Warburg effect’), with this pathway also providing precursors for protein, nucleotide and lipid biogenesis [12].

Mitochondria play key roles in cellular energy and metabolism, and in apoptosis, with mitochondrial abnormalities have been recognized in cancer [13]. VDAC1, a multi-functional channel, lies in the mitochondrial outer membrane and forms a pathway for the exchange of metabolites between mitochondria and cytosol, thereby regulating mitochondrial metabolic function and energy production [14,15,16,17]. VDAC1 also contributes to cancer cell metabolism via its binding to HK [15,17,18,19,20,21]. VDAC1-bound HK allows the coupling of mitochondrially-generated ATP to glucose phosphorylation, thus regulating glycolytic flux with the TCA cycle and ATP synthesis to balance the energy and metabolite requirements of tumor cells [15,17,21]. However, VDAC1 also directly contributes to mitochondria-mediated apoptosis by participating in the release of mitochondrial pro-apoptotic proteins to the cytosol (e.g. cytochrome *c* (Cyto *c*), apoptosis-inducing factor (AIF), second mitochondria-derived activator of caspases/Direct IAP-binding protein (SMAC/Diablo), [17,22] and by interacting with apoptosis regulatory proteins, such as members of the Bcl-2 family of proteins [18,23,24,25] and HK [18,19,20,21,26,27]. The relationship between the expression level of these energy and metabolic homeostasis- and apoptosis-associated proteins to CLL state and sensitivity to treatment was not previously addressed.

In this study, we profiled the expression of various proteins, including apoptosis and metabolic modulators, in peripheral blood mononuclear cells (PBMCs) isolated from CLL patients and healthy donors, in order to identify potential early biomarkers for CLL detection and guide treatment. Using liquid chromatography high-resolution mass spectrometry (LC-HR-MS/MS) analysis, we discovered 1,360 differentially expressed proteins in CLL patients compared to healthy donors, several of which had been previously reported to be associated with cancer, yet four of which were identified as being associated with any cancer, including CLL, for the first time. Many of these proteins are involved in mitochondrial metabolism, apoptosis, RNA splicing and translation. We demonstrate that besides increased expression of the anti-apoptotic protein Bcl-2, VDAC1, and the apoptosis-related proteins MAVS (mitochondrial anti-viral signaling protein, also known as IPS-1, Cardif or VISA [28]), AIF, and SMAC/Diablo, are also markedly over-expressed in lymphocytes of CLL patients. Furthermore, we could identify several proteins allowing for distinction between patients in a stable disease state and those transferred to anti-cancer treatments. The proteins identified in this study can thus serve as potential biomarkers for CLL diagnosis, predicting treatment effectiveness, new targets for cancer therapy and for defining optimal therapeutic interventions for patients, forming the basis for personalized therapy.

## Materials and Methods

### Materials

Dulbecco’s modified Eagle’s medium (DMEM) supplemented with L-glutamine was purchased from Gibco (Grand Island, NY). Fetal calf serum, penicillin-streptomycin, sodium pyruvate and non-essential amino acids were purchased from Biological Industries (Beit Haemek, Israel) Anti-actin monoclonal antibodies were from Millipore (Billerica, MA). Goat polyclonal anti-HK-I (sc-6517) and anti-HK-II (sc-6521) antibodies were obtained from Santa Cruz Biotechnology (Santa Cruz, CA). Rabbit polyclonal anti-BAX (PC66) and mouse monoclonal anti-Bcl-2 (OP60) antibodies were obtained from Calbiochem (Billerica, MA). Rabbit polyclonal anti-AIF (AF1457) antibodies were purchased from R&D Systems (Minneapolis, MN). Rabbit polyclonal anti-MAVS (ab-56230), rabbit polyclonal anti-VDAC1 (ab15895), rabbit polyclonal anti-SMAC/Diablo (ab-8115), anti-PPWD1 (ab-126710), anti-SLC25A1 (ab-99168), anti-DHRS4 (ab-68095), anti-UBE3A (ab-126765) and Cy2-conjugated anti-rabbit antibodies were obtained from Abcam (Cambridge, UK). Monoclonal anti-CD19 and CD5 antibodies were obtained from BD Bioscience (San Jose, CA). Horseradish peroxidase (HRP)-conjugated anti-mouse, anti-rabbit and anti-goat antibodies were from KPL (Gaithersburg, MD).

### Patients

Thirty one CLL patients from Soroka University Medical Center (14 males and 17 females with a median age of 69 years) were studied. CLL diagnosis was based on clinical examination, peripheral blood count and immuno-phenotyping (S1 Table). Patients were not receiving any disease treatment, with 68% being at stage 0–1, 6% at stage 2 and 26% at stage 3–4. The research was approved by the Soroka University Medical center Advisory Committee on Ethics in Human Experimentation.

All of the experiments were conducted in accordance with national laws and regulations, the ethical principles set forth in the Declaration of Helsinki and with good clinical practice as described in the ICH guidelines. Written informed consent was obtained from all participants prior to entry into the study. All subjects received a copy of their signed and dated informed consent form.

### Isolation of PBMC and CD19-positive cells

PBMCs were isolated from venous blood of CLL patients by Ficoll-Paque PLUS (GE Healthcare) density gradient centrifugation. After informed consent, venous blood (10–20 ml) was drawn from CLL patients or from normal adult donors. Blood was collected into heparin tubes and was diluted 1:1 with balance solution composed of solution A (1% D-glucose, 50 mM CaCl_2_, 0.98 mM MgCl_2_, 5.4 mM KCl and 0.145 M Tris-HCl, pH 7.6) and solution B (0.14 M NaCl) in a 1:9 ratio. The resulting mix was carefully layered on Ficoll-Paque Plus (10 ml of diluted blood on 15 ml Ficoll) in 50 ml conical tubes and centrifuged (400xg, 18–20°C, 40 min). The fine layer of mononuclear cells was transferred to a new centrifuge tube, washed thrice with balance solution and resuspended in culture medium appropriate to the application. The proportion of cancerous B cells in the total PBMC pool was analyzed by antibody-based detection of CD19/CD5 double positive cells and flow cytometry (Beckton-Dickinson, San Jose, CA).

CD19-positive cells were isolated from PMBCs using a magnetic bead-based method following the manufacturer's instructions, with minor modifications. Briefly, PMBCs (2.5x10^7^ cells) were centrifuged (300g, 10 min), resuspended in 200 μl of buffer A (PBS, pH, 7.2, 2 mM EDTA, 2% FBS), labeled with 50 μl of CD19 micro beads (Cat. No: 130-050-301, MACS Miltenyi biotech), mixed and incubated for 15 minutes at 4°C. The cells were washed with buffer A, centrifuged, resuspended in buffer A and subjected to magnetic separation with LS columns and a Midi MACS separator from MACS Miltenyi Biotech.

### Cell culture and transfection

MEC-1 cells were grown in DMEM supplemented with 10% FBS, 1 mM L-glutamine, 100 U/ml penicillin, and 100 μg/ml streptomycin at 37°C and 5% CO_2_. siRNAs were synthesized by Genepharma. Two siRNA sequences were used for each gene (sense (S) and anti-sense (AS) sequences). For siRNA-DDX46 (1) S: 1104–5′AAGUUGAUCUUCAGACAGCCC UU3′-1126 and AS: 5′AAGGGCUGUCUGAAGAUCAACUU3′ and (2) S: 1320–5’AAUCCUGG GUCC AGUGUGGAAUU3′-1342 and AS: 5′AAUUCCACACUGGACCCAGGAUU3′. For siRNA-AK3 (1) S: 5995’CAGAGACGGUUAUCAAGAGACUAAA3’-623 and AS: 5’UUUAGUCUCUUGAUAACCGUCUCUG3’ and (2) S: 704–5’CCAACAAGAUUUGGCC CUAUGUAUA3’-728 and AS: 5’UAUACAUAGGGCCAAAUCUUGUUGG3’. The scrambled siRNA used were S: 5'GCAAACAUCCCAGAGGUAU3' and AS: -5'AUACCUCUGGGAUGUUUGC3'

Cells were seeded in DMEM supplemented with 5% FBS (150,000 cells/well) in 6-well culture dishes to 40–60% confluence and transfected with 50 nM of the siRNA using the transfection reagent GeneTran III,(Biomiga) according to the manufacturers’ instructions.

### Quantitative real-time PCR (qRT-PCR)

Total RNA was isolated from control-, siRNA-Scrambled-, siRNA-AK3-1- and 2-, siRNA-DDX46-1- and 2-, AK3 1 and 2 siRNA- and DDX46-1 and 2 siRNA-transfected cells using an RNeasy mini kit (Qiagen) according to the manufacturer's instructions. Total RNA quality was analyzed using the Agilent RNA 6000 nano kit. The RNA integrity values obtained for total RNA were 8–10. Complementary DNA was synthesized from 1 μg total RNA using a Verso cDNA synthesis kit (Thermo Scientific). Real-time fluorescent RT-PCR was performed using specific primers for DDX46 (forward GCCCCAAACCAATTAAATCCTG and reverse CAATGCCAATCAAATCTCGTCC) and AK3 (forward TTACTGCTCGCTGGATTCATC and reverse GTCTCTTGATAACCGTCTCTGG) (KiCqStart Primers; Sigma Aldrich) in triplicate, using Power SYBER green master mix (Applied Biosystems, Foster City, CA). The levels of the target genes were normalized relative to β-actin mRNA levels assessed using appropriate primers (forward ACTCTTCCAGCCTTCCTTCC, reverse TGTTGGCGTACAGGTCTTTG). Samples were amplified by a 7300 Real Time PCR System (Applied Biosystems) for 40 cycles using the following PCR parameters: 95°C for 15 seconds, 60°C for 1 minute, and 72°C for 1 minute. Copy numbers for each sample were calculated by the CT-based calibrated standard curve method. The means fold-change (± SEM) of the three replicates were calculated.

### SRB assay for cell proliferation

Forty-eight or 96 h post-transfection with siRNA, cells were washed twice with PBS, fixed with 10% trichloroacetic acid for 1–2 h, and subsequently stained with SRB. SRB was extracted from the cells using 100 mM Tris-base and absorbance at 510 nm was determined using an Infinite M1000 plate reader (Tecan, Männedorf, Switzerland).

### Gel electrophoresis and immunoblotting

PBMCs isolated from CLL patients or healthy donors were resuspended in lysis buffer (50 mM Tris-HCl, pH 7.5, 150 mM NaCl, 1 mM EDTA, 1.5 mM MgCl_2_, 10% glycerol, 1% Triton X-100, a protease inhibitor cocktail (Calbiochem)), sonication, centrifuged (17,500xg, 15 min, 4°C) and samples (10–40 μg of protein) were subjected to SDS-PAGE. For immunostaining, gels were electro-transferred onto nitrocellulose membranes blocked with 5% non-fat dry milk and 0.1% Tween-20 in Tris-buffered saline and incubated overnight at 4°C with the different primary antibodies, followed by incubation with the relevant HRP-conjugated secondary antibodies (1 h) and developed using enhanced chemiluminescence (Biological Industries). Band intensities were analyzed by densitometry using Multi Gauge software (Fujifilm). Readings were normalized to the intensities of β-actin signals that served as a loading control.

### Assessment of mitochondrial mass

Cells were loaded with 20 nM MitoTracker Green (Molecular Probes) for 15 min at 37°C, with the fluorescence signal being unaffected by mitochondrial membrane potential, thereby providing a measure of mitochondrial mass.

### Proteomics analysis

PBMCs were solubilized in a lysis buffer (50 mM Tris-HCl, pH 7.5, 150 mM NaCl, 1 mM EDTA, 1.5 mM MgCl_2_, 10% glycerol, 1% Triton X-100, a protease inhibitor cocktail (Calbiochem)), followed by sonication and centrifugation (10 min, 600g). Protein concentration of each lysate was determined using a BCA assay. Samples were then subjected to in-solution tryptic digestion as follows. Proteins were first reduced by incubation with 5 mM DTT for 30 min at 60°C, followed by alkylation with 10 mM iodoacetamide in the dark for 30 min at 21°C. Proteins were then subjected to digestion with trypsin (Promega; Madison, WI) at a 1:50 trypsin:protein ratio for 16 h at 37°C. Following digestion, detergents were cleared from the samples using commercial detergent removal columns (Pierce, Rockford, IL), and desalted using solid-phase extraction columns (Oasis HLB, Waters, Milford, MA). Digestions were stopped by addition of trifluroacetic acid (1%). The samples were stored at -80˚C until analysis.

### LC-HR-MS/MS

For LC-HR-MS/MS, ULC/MS grade solvents were used for all chromatographic steps. Each sample was separated using split-less nano-ultra performance liquid chromatography columns (10 kpsi nanoAcquity; Waters, Milford, MA). The mobile phase was: (A) H_2_O and 0.1% formic acid, and (B) acetonitrile and 0.1% formic acid. Desalting of the samples was performed online using a reverse-phase C18 trapping column (180 μm internal diameter, 20 mm length, 5 μm particle size; Waters). The peptides were then separated using a T3 HSS nano-column (75 μm internal diameter, 250 mm length, 1.8 μm particle size; Waters) at 0.3 μL/min. Peptides were eluted from the column into the mass spectrometer using the following gradient: 4% to 35% (B) for 150 min, 35% to 90% (B) for 5 min, maintained at 90% for 5 min and then back to initial conditions. The nano-UPLC was coupled online through a nano-ESI emitter (10 μm tip; New Objective; Woburn, MA) to a quadrupole Orbitrap mass spectrometer (Q Exactive, Thermo Scientific) using a FlexIon nanospray apparatus (Proxeon). Data was acquired in the DDA mode, using a Top12 method [29]. Raw data was imported into Expressionist software (Genedata) [30,31]. The software was used for retention time alignment and peak detection of precursor peptide intensities. A master peak list was generated from all MS/MS events and sent for database searching using Mascot v2.4 (Matrix Sciences). Data was searched against a database containing forward and reverse human protein sequences from UniprotKB-SwissProt, and 125 common laboratory contaminants, totaling 20,304 entries. Fixed modification was set to carbamidomethylation of cysteines, while variable modification was set to oxidation of methionines. Search results were then imported back to Expressionist for annotation of detected peaks. Identifications were filtered such that the global false discovery rate was a maximum of 1%. Protein abundance was calculated based on the three most abundant peptides [32].

Two independent LC-HR-MS/MS analyses of healthy donors and CLL patients were performed, with six healthy donors and nineteen CLL patients, and five age-matched healthy donors and nine CLL patients, were used in the first and second analysis, respectively. Proteins with at least one unique peptide identified were used for further analysis.

### Statistics and bio-informatics analyses

Means ± SEM of results obtained from indicated independent experiments are presented. The level of significance of differences between control (healthy) and experimental (CLL patients) groups was determined using the non-parametric Mann-Whitney U test. A difference was considered statistically significant when the *P* value was deemed <0.05 (*), < 0.01 (**) or <0.001 (***). Statistics for data analysis were computed using the SPSS statistical package, version 17.0. A non-parametric receiver operating characteristic (ROC) curve was plotted and the area under the ROC curve (AUC) was estimated, indicating the probability of the protein to be a valid diagnostic marker, with values ranging from 0 to 1, where 1 indicates a perfect marker [33]. LC-HR-MS/MS data were imported into Partek Genomics Suite software (Partek, St. Louis, MO) and difference between expression levels of the proteins in the different groups was calculated using the one-way analysis of variance (ANOVA). Functional enrichment analysis of significantly different proteins was performed using DAVID bioinformatics resources v6.7 [34].

Kaplan-Meier survival curves were constructed to compare survival between CLL patients with high and low expression levels of the indicated protein. P< 0.05 was taken to indicate a statistically significant difference.

## Results

In this study, PBMCs isolated from healthy donors and CLL patients receiving no disease treatment were analyzed using LC-HR-MS/MS and immunoblotting to explore possible CLL biomarkers and disease progression predication. Many potential biomarkers for CLL were identified in this manner, yet we focused on the correlation of CLL occurrence with the expression profiles of several metabolism- and apoptosis-related proteins, as well as other highly specific biomarkers that were identified. Special attention was given to VDAC1, a regulator of cellular energy, metabolism and apoptosis. The results offer novel biomarkers for CLL diagnosis and treatment guidance.

### Mass spectrometry analysis of protein profile of PBMCs from CLL patients and healthy donors

**Fig 1:**
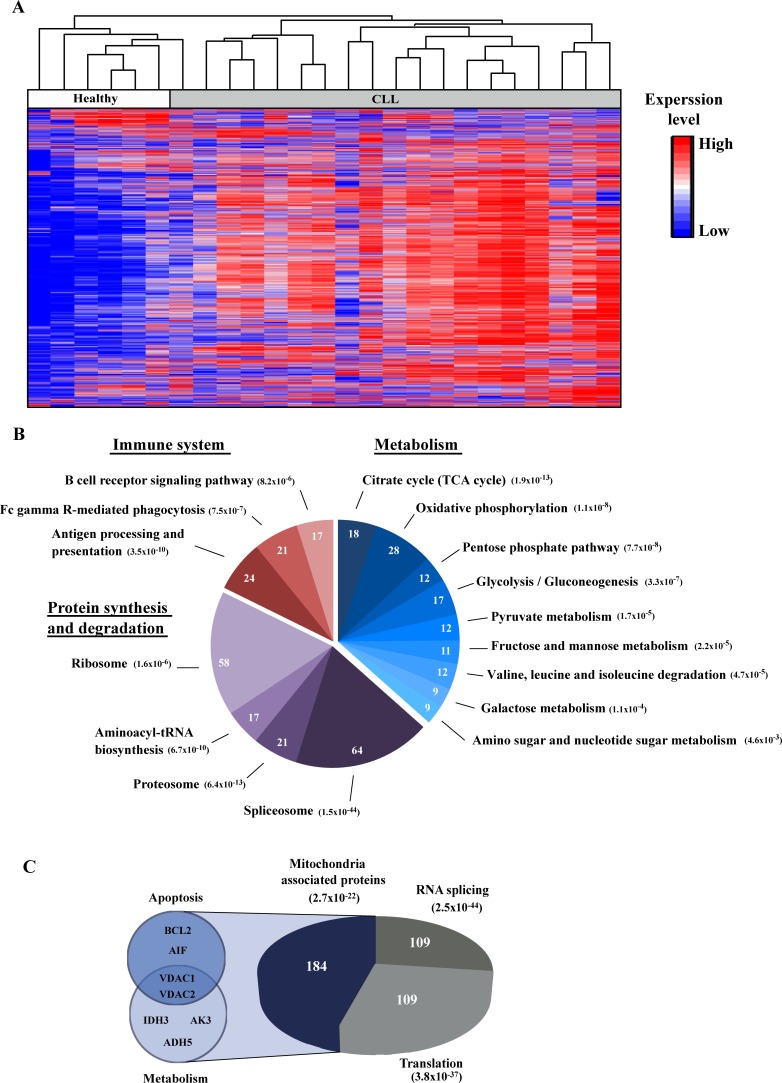
Hierarchical clustering and functional analysis of proteins expression in PBMCs obtained from healthy controls and CLL patients. The protein expression profiles of PBMCs obtained from healthy donors and CLL patients were analyzed by LC-HR-MS/MS as described under Materials and Methods. **A.** Hierarchical clustering based on the expression pattern of all 2,441 detected proteins with at least 1 unique peptide is presented. Healthy (white) and CLL (grey) are indicated. The color scale of the standardized expression values is shown on the right. **B.** Significantly enriched pathways associated with differentially expressed proteins in CLL, with their enrichment p-value, are presented. The number of proteins related to each pathway is indicated inside the chart. **C**. Significantly enriched functional groups based on the DAVID functional analysis are presented, with their enrichment p-value. The number of proteins related to each functional group is indicated inside the chart. Small pies on the left indicate apoptosis- and metabolism-related proteins of interest.

To identify the proteins whose expression levels were modified in CLL-derived PBMCs in comparison to PBMCs from healthy individuals, LC-HR-MS/MS analysis was performed. PBMC samples were collected from six healthy individuals and nineteen CLL patients. Hierarchical clustering based on the protein expression pattern clearly allowed for distinction between the healthy donors and the CLL patients (Fig 1A), with the expression level of 1,360 proteins being changed (fold change (FC) **≥|**2**|** and p-value < 0.01, of which 118 with a FC ≥100).

Next, functional analysis of proteins differentially expressed between healthy donors and CLL patients was performed using the DAVID tool [34] based on the KEGG [35,36,37,38] databases. The analysis revealed enrichment for biological pathways related to metabolism, the immune system and protein synthesis and degradation (Fig 1B), as well of proteins belonging to the mitochondria, RNA splicing and translation functional groups (or associated functions) (Fig 1C). Of interest is the high enrichment of mitochondria-related functions in both analyses, which included proteins involved in apoptosis and metabolism. Similarly, enrichment of RNA splicing proteins, where mutations were shown to be indicative of high-risk in CLL patients, was seen [39,40]. These results indicate the validity of the MS analysis, which was thus used for further analysis.

### Identification of novel bio-markers for CLL

To further select proteins for continued focus, we repeated the LC-HR-MS/MS analysis using 14 samples (from five age-correlated healthy donors and nine CLL patients) that included new as well as previously analyzed samples. This narrowed our focus to 59 proteins that presented a clear common expression pattern in both LC-HR-MS/MS analyses. Changes were seen in the expression of a panel of proteins associated with metabolism and apoptosis, pre-mRNA processing, organelle biogenesis and more (Fig 1B and 1C). Moreover, some of these proteins were previously found to be mutated in CLL or proposed as markers of CLL or other cancers, while others were not previously connected to cancer.

Selected proteins were arranged into three groups (Table 1A–1C). The first group included five proteins that have previously been identified as being mutated in CLL or proposed as CLL diagnosis and prognosis markers (Table 1, group A).

**Table 1:** List of selected proteins differentially expressed between healthy donors and CLL patients identified by LC-HR-MS/MS.

Protein (UniProt accession)	Fold change/P value		Proposed function (cell localization)	Relation to cancer
**LC-HR-MS/MS analysis:**	**1**^**st**^	**2**^**nd**^	**Group A—CLL associated proteins**	
BCL-2- B-cell lymphoma 2 (P10415)	24.5 1.1x10^-7^	6.4 3.4x10^-3^	Suppresses apoptosis (mitochondria)	Over-expressed in CLL [41]
IGHD- immunoglobulin heavy constant delta (P01880)	44.2 2.3x10^-6^	>1000 2.1x10^-13^	Major antigen receptor on the surface of B-cells (secreted, plasma membrane)	Mutation in IGHD used in prognosis of CLL [42]
KSYK- spleen tyrosine kinase (P43405)	6.8 7.5x10^-6^	4.0 2.4x10^-4^	Mediates signal transduction (plasma membrane, cytoplasm)	Enhanced expression in CLL [43]
NFKB2- NF-kappa-B p100 (Q00653)	21.6 3.1x10^-6^	93.3 9.9x10^-3^	Transcription factor, involves in inflammation, immunity, differentiation, tumorigenesis and apoptosis (nucleus, cytoplasm)	NF-kappa B2/p100 induces Bcl-2 expression in CLL [44]
CD74- HLA class II histocompatibility antigen gamma chain (P04233)	5.1 1.7x10^-4^	5.1 4.5x10^-5^	MHC class II antigen processing (plasma membrane)	CD74 expression correlates with ZAP70 in CLL [45]
**LC-HR-MS/MS analysis:**	**1**^**st**^	**2**^**nd**^	**Group B–Cancer associated proteins**	
VDAC1- voltage dependent anion channel 1 (P21796)	5.3 2.8x10^-5^	4.8 4.8x10^-4^	Ions and metabolites transport. Involves in apoptosis (OMM, plasma membrane)	Over expressed in different tumor tissues [46]
VDAC2- voltage dependent anion channel 2 (P45880)	3.1 1.4x10^-3^	4.5 7.4x10^-4^	Transport of ions and metabolites (OMM)	High levels in liver cancer [47]
AIF- apoptosis inducing factor	10.6 3.6x10^-9^	___	Initiator of apoptosis (mitochondria, in apoptosis translocates to nucleus)	Overexpressed in cancer [48,49,50]
IDH3A- isocitrate dehydrogenase 3 (P50213)	2.6 7.1x10^-3^	6.8 9.6x10^-5^	TCA cycle (mitochondria)	IDH1, IDH2 mutated in leukemia and brain tumors [51,52]
BRI3B- BRI3-binding protein (HCCRBP-1) (Q8WY22)	5.8 2.9x10^-5^	389.4 2.7x10^-3^	Outer mitochondrial membrane	Induce tumorigenesis through p53 stabilization [53]
PPWD1- peptidylprolyl isomerase domain and WD repeat containing 1 (Q96BP3)	35.7 9.7x10^-5^	105.4 7.7x10^-3^	Accelerates the folding of proteins, possibly involved in pre-mRNA splicing (nucleus)	Marker for pancreatic cancer [54]
GELS- gelsolin (P06396)	-3.3 4.3x10^-3^	-5.0 4.0x10^-3^	Actin modulating protein (secreted, cytoplasm)	Down regulated in leukemia, cervical and breast cancer [55,56]
LTBP1-latent TGFβ binding protein 1 (Q14766)	-4.7 1.6x10^-5^	-6.2 7.9x10^-3^	Associates with pro-TGFβ complex. Modulates TGFβ activity (secreted)	Increased expression in human glioma cells [57]
**LC-HR-MS/MS analysis:**	**1**^**st**^	**2**^**nd**^	**Group C—Newly identified CLL associated proteins**	
ADH5- alcohol dehydrogenase 5 (P11766)	2.8 8.1x10^-4^	3.5 7.8x10^-4^	Metabolism of alcohols and aldehydes (cytoplasm)	**No direct association reported**
AK3- adenylate kinase 3 (Q9UIJ7)	29.9 3.0x10^-5^	4.1 1.9x10^-3^	Maintaining the homeostasis of cellular nucleotides (mitochondria)	**No direct association reported**
DDX46- DEAD box protein 46 (Q7L014)	9.1 1.9x10^-5^	201.4 1.2x10^-3^	Pre-mRNA processing (nucleus)	**No direct association reported**
AP3B1- AP-3 complex subunit beta-1 (O00203)	9.6 1.7x10^-4^	698.8 8.5x10^-5^	Biogenesis of late endosomal/ lysosomal structures (Golgi membrane)	**No direct association reported**

Two independent LC-HR-MS/MS experiments were performed as described in the Materials and Methods section. From each experiment, differentially expressed proteins (p-value <0.01, FC ≥|2**|**) were filtered and proteins differentially expressed in both experiments were selected. Proteins of relevance to CLL or with potential as biomarkers are listed. For each protein, the name, fold change and p-value in each experiment, as well as its function, subcellular localization and relevance to cancer are indicated. Proteins were divided into three groups based on their known association to CLL, relation to metabolism or potential as metabolism-unrelated biomarkers for CLL.

A second group included several proteins whose expression levels were significantly different between healthy and CLL patients that were previously reported to be associated with cancers, other than CLL (Table 1, group B). These include BRI3BP, shown to induce tumorigenesis through p53 stabilization [53], a putative peptidylprolyl isomerase (PPIase, PPWD1) that accelerates the folding of proteins and may be involved in pre-mRNA splicing [54], and gelsolin, expression levels of which were decreased several fold, as found for several cancer types, including U937, human myelomonocytic leukemia cell line, cervical and breast cancers. Gelsolin was shown to inhibit apoptosis via interaction with VDAC1 [58]. LTBP1 is associated with pro-TGFβ complex and modulates TGFβ activity [57,59]. LTBP1 expression level were decreased by about 5-fold in CLL patients, but were reported to increase in human glioma cells [57]. This group also included proteins associated with metabolism and apoptosis, included proteins that were reported in other cancers, including liver and brain tumors, but not CLL, such as VDAC1, VDAC2, AIF and other mitochondrial proteins associated with metabolism and apoptosis regulation (Fig 1C and Table 1, group B).

**Fig 2:**
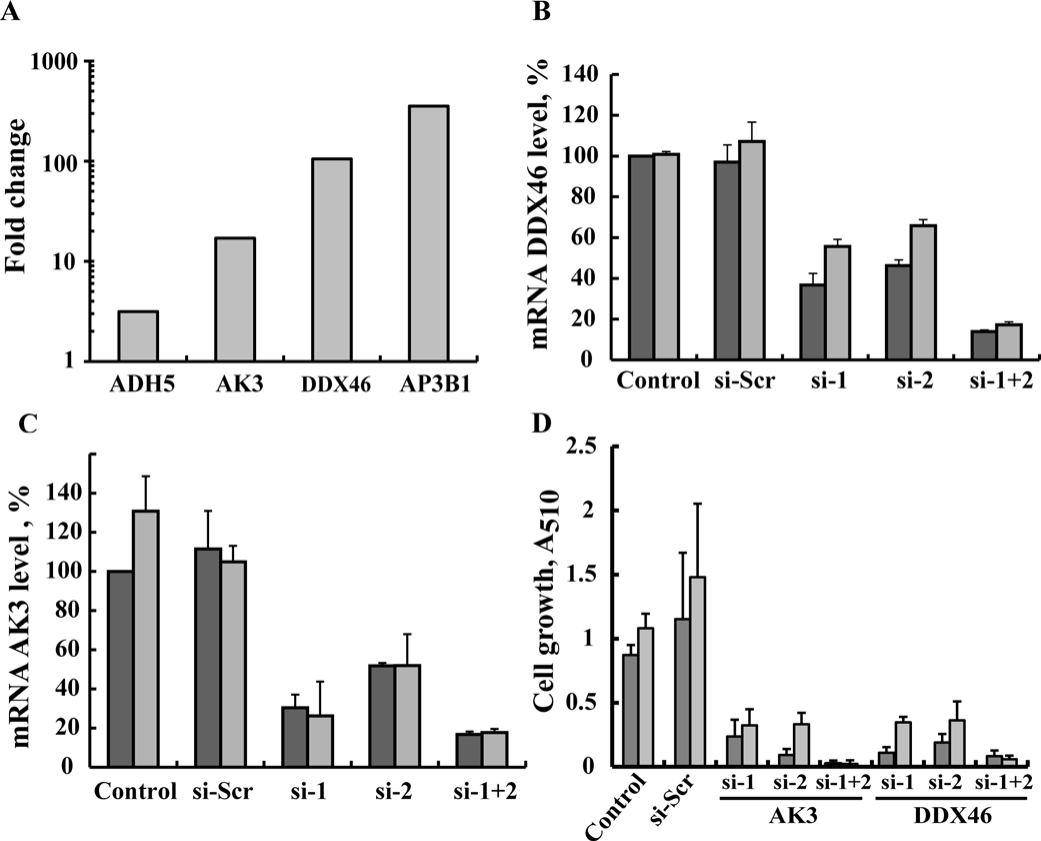
siRNA silencing of AK3 or DDX46 expression inhibits cell growth. (**A**) Differential expression of the 4 proteins not previously connected to any cancer between healthy and CLL individuals. (**B-D**) MEC-1 cells were transfected with (50 nM) scrambled siRNA (si-Scr,), one of the 2 different siRNAs against AK3 (siAK3 1 or 2), or against DDX46 (si-DDX46 1 or 2), a combination of siAK3 1 and 2 or a combination of siDDX46 1 and 2 and, at the indicated time, were analyzed for AK3 and DDX46 mRNA levels by RT-PCR (**B,C**) or analyzed for cell growth using the SRB method (n = 3) (**D**).

A third group included proteins whose expression was increased 3 to over 100-fold in CLL but were not previously connected to any cancer (Table 1, group C, Fig 2A). These include AP3B1, involved in the biogenesis of late endosomal/lysosomal structures [60], DDX46 RNA helicase, involved in pre-mRNA processing [61], alcohol dehydrogenase 5 (ADH5) involved in the metabolism of alcohols and aldehydes [62], and the mitochondrial protein adenylate kinase 3 (AK3), involved in maintaining the homeostasis of cellular nucleotides [63].

To verify the importance of these proteins to CLL cancer cells, we tested the effects of their silencing on cell viability and growth using specific siRNA. Owing to limited access to CLL samples and their low stability, we instead used MEC-1 cells derived from B-chronic lymphocytic leukemia in prolymphocytoid transformation [64] to analyze the effects of siRNA-mediated down-regulated expression of DDX46 and AK3 on cell viability (Fig 2D). While two sets of siRNA were designed and tested for each of four proteins, only those designed for DDX46 and AK3 effectively decreased their expression (Fig 2B and 2C). Cells were transfected with scrambled siRNA or siRNA specific to DDX46 or AK3 and cell growth was analyzed using the SRB method. Each of the designed siRNAs was highly effective in decreasing cell growth up to 80% 48 h post-transfection, with the combination of both siRNAs being more active in decreasing both mRNA levels and cell growth (over 90%) (Fig 2B–2D). These effects required only nanomolar concentrations (50 nM) and persisted 96 h post-transfection (Fig 2B–2D). The decrease in cell growth was due to a decrease in the number of cells and not due to cell death (data not shown).

Finally, to further test the prognostic value of AP3B, ADH5, DDX46 and AK3 proteins in CLL, we used Kaplan-Meier survival analysis on public available gene expression data a set of CLL patients in which the percent of patients surviving with high mRNA levels was compared to the survival of patients with low mRNA levels. Significant difference between the two patient groups was found for DDX46 (p = 0.00097) but not for AP3B (P = 0.469), ADH5 (P = 0.156) and AK3 (P = 0.065) (S1 Fig). Data analysis was performed with the DRUGSURV bioinformatics analysis tool (http://www.bioprofiling.de/GEO/DRUGSURV).

### Expression of VDAC1 and apoptosis/metabolic modulators is highly elevated in most CLL patients

Since avoidance of apoptosis is considered the hallmark characteristic of CLL [6,7,8,9,65], we turned our attention to analyzing VDAC1 and other key apoptosis-modulating proteins whose expression was altered in CLL patients, as demonstrated in the LC-HR-MS/MS analysis. To validate our MS analysis, we used protein-specific antibodies and large PBMC samples from healthy donors and CLL patients.

**Fig 3:**
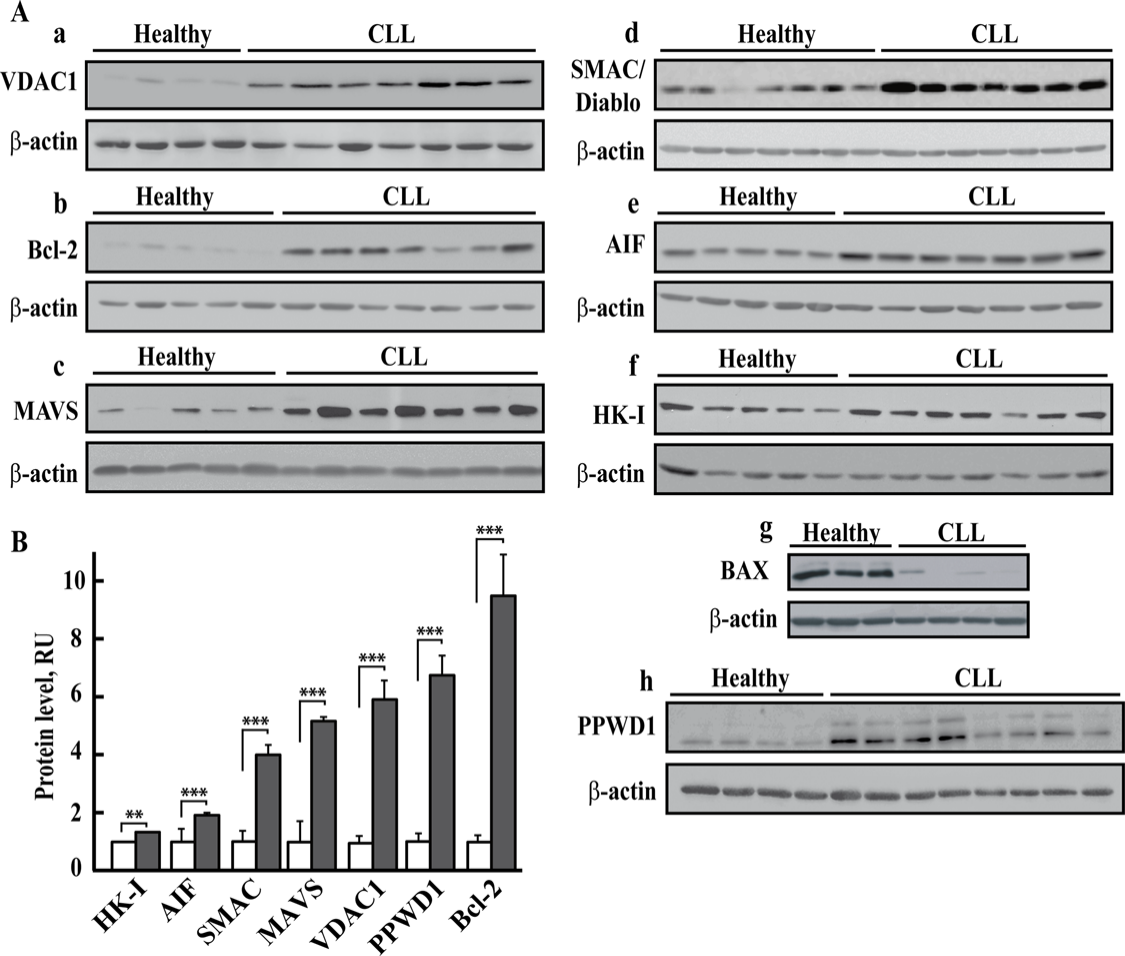
Over-expression of Bcl-2, VDAC, AIF, MAVS, SMAC/Diablo and PPWD1 in PBMCs from CLL patients. Immunoblot analysis of cell lysates of PBMCs derived from CLL patients (P) and healthy donors (H) probed with antibodies directed against VDAC1 [A**a**, n = 28 (P), 20 (H)], Bcl-2 [A**b**, n = 28 (P), 20 (H)], MAVS [A**c**, n = 28 (P), 19 (H)], SMAC/Diablo [A**d**, n = 21 (P), 15 (H)], AIF [Ae, n = 17 (P), 16 (H)], HK-I [A**f**, n = 28 (P), 20 (H)], BAX [A**g**, n = 6 (P), 6 (H)], PPWD1 [A**h**, n = 16(P), 10(H)] or β-actin. Representative immunoblots (**A**) and quantitative analysis (mean ± SEM) (**B**) of protein levels of healthy donors (white) and CLL patients (grey) of these and other samples are presented. For each sample, three independent immunoblots were performed. A difference between healthy and CLL groups was considered statistically significant when P < 0.001 (***) or P < 0.01 (**), as determined by the Mann-Whitney test.

The expression levels of VDAC1 and several apoptosis-related proteins in PBMCs obtained from 31 CLL patients and 26 healthy donors were assessed using antibodies specific to each protein (representative immunoblots are shown in Fig 3A). The results clearly demonstrate marked increases in Bcl-2, VDAC1, MAVS, AIF and SMAC/Diablo protein levels and a slight increase in the level of HK-I. Protein levels were normalized to that of β-actin by band intensity quantitative analysis of three independent immunoblots, and are presented as means ± SEM (Fig 3B).

Specifically, VDAC1 was found to be substantially over-expressed by 6.2-fold in PBMCs from CLL patients, as compared with healthy donors (Fig 3Aa and 3B). Likewise Bcl-2 protein levels were markedly higher (9.6-fold) in PBMCs obtained from CLL patients (Fig 3Ab and 3B). MAVS was over-expressed on average by 5.2-fold in PBMCs from CLL patients, as compared with healthy donors (Fig 3Ac and 3B). This is the first demonstration of VDAC1 and MAVS over-expression in leukemia. In line with published data [8,10,11,66], The expression of VDAC1, MAVS and was also examined in CLL PBMCs from CLL patients and healthy donors by immunocytochemistry (S2 Fig). Unexpectedly, the mitochondrial pro-apoptotic proteins AIF [67] and SMAC/Diablo [68] were also over-expressed (1.9- and 4-fold on average, respectively) in PBMCs derived from CLL patients, as compared with healthy donors (Fig 3Ad and 3Ae, respectively and 3B).). This is the first demonstration of AIF and SMAC/Diablo over-expression in leukemia.

To assess HK levels, we used several commercially available antibodies directed against HK-I or HK-II, most of which displayed non-isoform-specific reactivity. In CLL patients, HK-I levels were slightly increased (1.35-fold) (Fig 3Af and 3B), while assessment of HK-II levels proved inconclusive (not shown). The levels of the pro-apoptotic protein Bax were very low in CLL patients, being less than 10% of those found in healthy donors (Fig 3Ag and 3B).

The over-expression of Bcl-2, VDAC1 and AIF in CLL as revealed by immunoblotting was in agreement the LH-HR-MS/MS results (Table 1 group A,B).

We also selected PPWD1, over-expressed in CLL patients (35.7 fold, P = 9.7x10^-5^ and >100 fold, P = 7.7x10^-3^, from the newly identified CLL associated proteins group (Table 1, group B). Immunoblotting confirmed that PPWD1 was over-expressed in CLL patients (Fig 3Ah and 3B).

The observed increases in protein levels were not due to increased mitochondrial numbers/mass, since analysis using MitoTracker green, a dye that localizes to mitochondria regardless of mitochondrial membrane potential, revealed similar levels of mitochondria in PBMCs derived from either CLL patients or healthy donors (S3 Fig).

**Fig 4:**
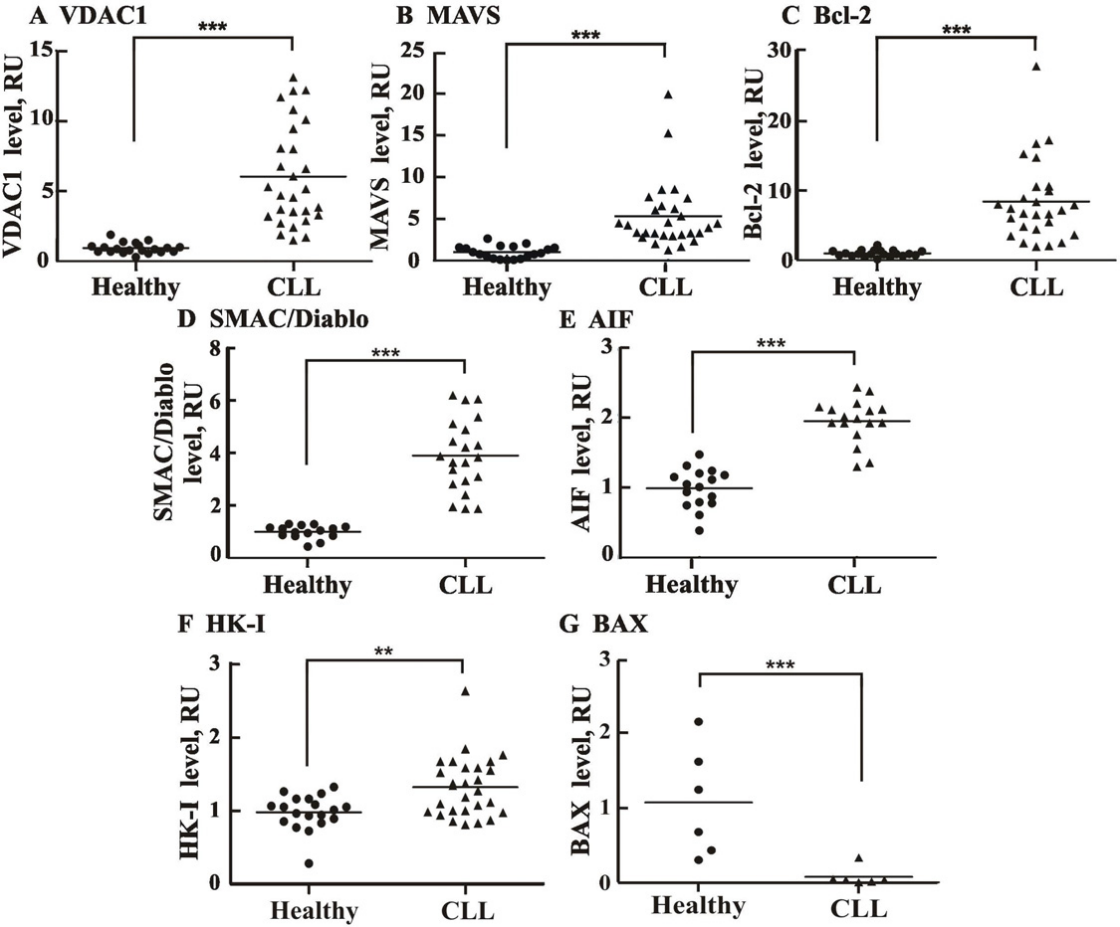
Comparison of apoptosis-related proteins in CLL patient- and healthy donor-derived PBMCs. Scatter plots display the expression levels of VDAC1 (**A**), MAVS (**B**), Bcl-2 (**C**), SMAC/Diablo (**D**), AIF (**E**), HK-I (**F**) and Bax (**G**), for each control subject and CLL patient, as analyzed in Fig 2. Statistics were calculated with GraphPad Prism software. Horizontal lines represent mean values for each group. A difference between the healthy donor and CLL patient groups was considered statistically significant when P < 0.001 (***) or P < 0.01 (**), as determined by the Mann-Whitney test.

Vertical scatter plots comparing the levels of VDAC1 and other proteins in individual healthy and CLL donors demonstrated significant differences between the two groups in each case (Fig 4A–4G). Eighty to over 90% of the CLL patients showed VDAC1, Bcl-2, SMAC/Diablo, MAVS and AIF levels higher than seen in all healthy donors. In some patients, Bcl-2 and VDAC1 levels were 30- and 14-fold higher than in healthy donors, respectively. Similarly, SMAC/Diablo and MAVS were 6- and 20-fold over-expressed in some patients, respectively.

### VDAC1 levels correlate with the levels of CD19+/CD5+ cancerous cells, SMAC/Diablo, and Bcl-2

**Fig 5:**
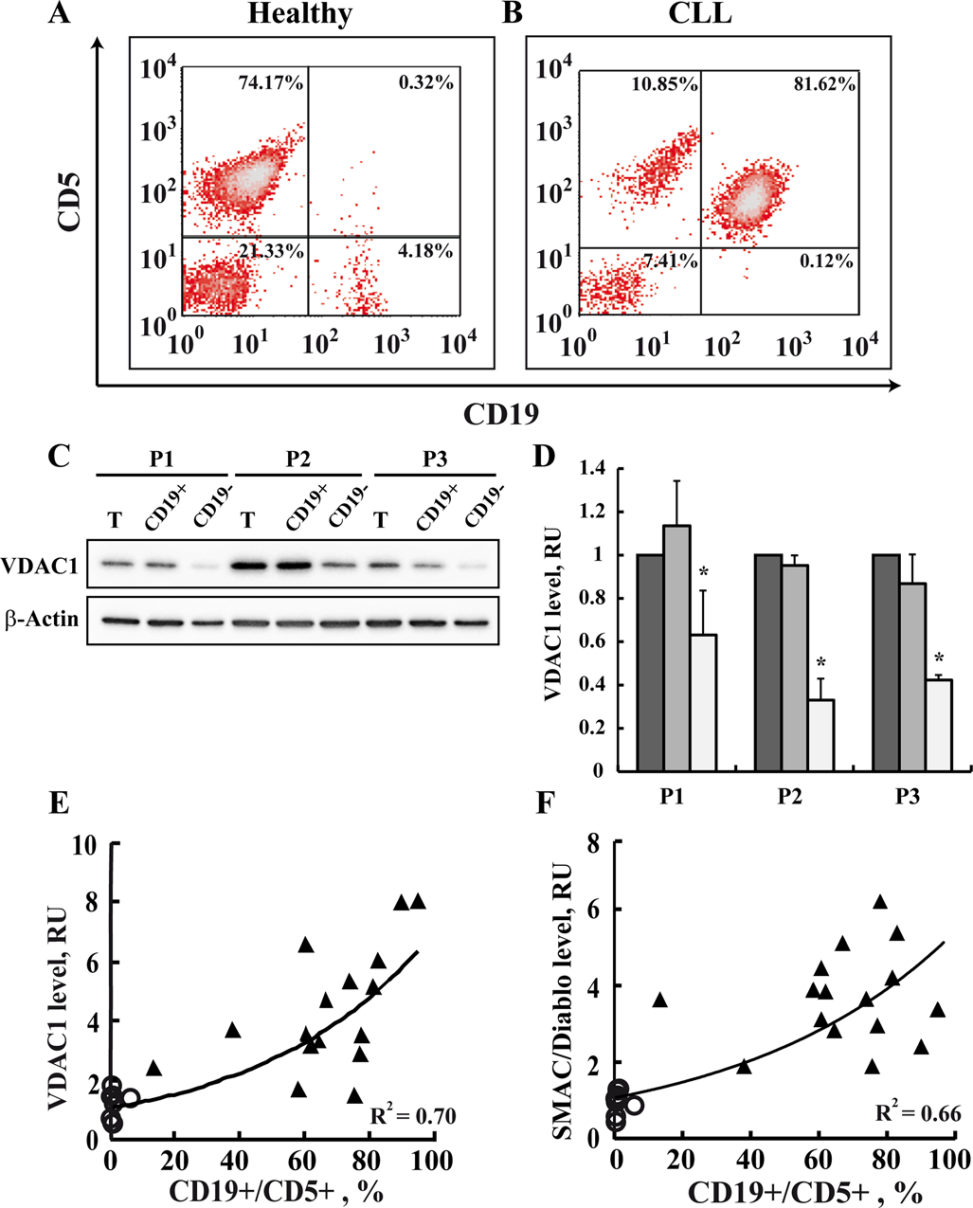
The VDAC1 expression level is correlated with the level of cancerous CD19+/CD5+ cells and apoptosis-related proteins. The percentages of CD19+/CD5+ cells in PBMCs isolated from representative healthy donor (n = 9) (**A**) or CLL patient (n = 16) (**B**) were determined using monoclonal antibodies directed to CD19/CD5, by flow cytometry analysis. CD19+/CD5+ cells represent cancerous CLL B lymphocytes. PBMCs obtained from 3 CLL patients (P (were subjected to CD19-positive cell separation using a magnetic bead-based method described in Materials and Methods. VDAC1 levels in PBMCs and their CD19-positive and -negative fractions were analyzed by immunoblotting using anti-VDAC1 antibodies (**C**). Quantitative analysis (mean ± SEM) (**D)** is presented. VDAC1 (**E**, R^2^ = 0.7) and SMAC/Diablo (F, R^2^ = 0.66) expression levels were determined as a function of the percentage of CD19+/CD5+ cells for each healthy donor (O) and CLL patient (▲). VDAC1 levels were assayed as described in the legend to Fig 3.

The relationship between VDAC1 levels and the relative amounts of CD19, a B lymphocyte marker, and CD5, a T-lymphocyte marker (CD19+/CD5+) cells (representing CLL cancerous cells) in PBMCs isolated from each patient was assessed using specific antibodies (Fig 5). Representative flow cytometric analyses of PBMCs from a single patient and a healthy donor demonstrated that about 82% of the patient’s cells were CD19+/CD5+ (Fig 5B), while less than 1% of such cells were found in PBMCs from a healthy donor (Fig 5A).

To demonstrate that VDAC1 levels were high in the cancerous cells, we separated CD19-positive cells using anti-CD19 antibodies conjugated to magnetic beads (Fig 5C). PBMCs isolated from three CLL patients presented 83, 86 and 92% CD19/CD5-positive cells (data not shown). The CD19 cell population represents cancerous cells as in healthy individuals, B lymphocytes comprise only 1–7% of the population [69]. VDAC1 levels in the CD19-positive and negative cells were analyzed by immunoblotting (Fig 5C and 5D). VDAC1 levels in CD19-positive cells were several-fold higher than in CD19-negative cells.

Next, the level of VDAC1 expression, plotted as a function of the percentage of CD19+/CD5+ cells for each sample (Fig 5E), showed that the VDAC1 level was positively correlated with the amount of CD19+/CD5+-expressing cancerous cells. This correlation further demonstrates that an elevation in the expression of VDAC1 was strongly associated with cancerous CLL cells. Similarly, positive correlation between the amount of CD19+/CD5+-expressing cells and SMAC/Diablo (Fig 5F), MAVS, Bcl-2 and AIF (R^2^ = 0.5–0.7, data not shown) expression levels was obtained.

VDAC1 levels were positively correlated with the apoptosis modulator proteins SMAC/Diablo and Bcl-2 over-expressed in CLL (S4A and S4B Fig). These results suggest that the VDAC1 expression level is associated with the expression of these apoptosis-modulating proteins.

### VDAC1 and apoptosis-modulating protein expression as a predictor of CLL

**Fig 6:**
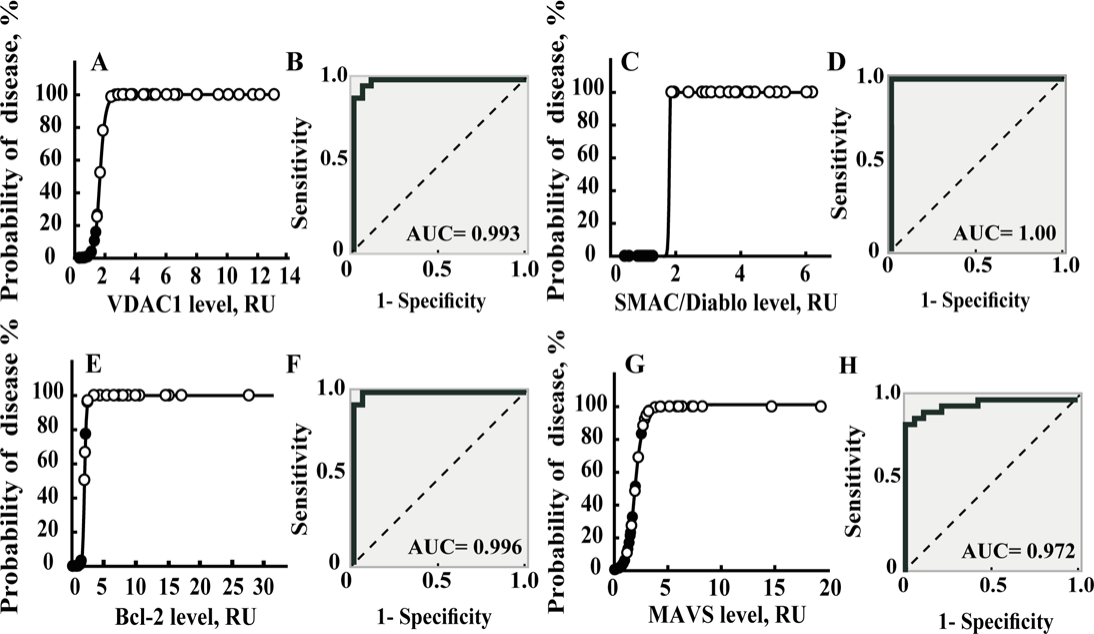
Binary logistic regression testing for specificity, sensitivity and overall CLL predication based on the relative expression of apoptosis-related proteins. Bivariance analysis was performed based on the relative expression of apoptosis-related proteins from Fig 3, considered as independent variables. Data was analyzed in terms of the sensitivity and specificity by assessing levels of apoptotic-regulating proteins based on a cut-off value of 0.5, using binary logistic regression analysis. Probability of disease is presented for healthy donor (●) and CLL patient (O) for VDAC1 (A), SMAC/Diablo (C), Bcl-2 (E) and MAVS (G). The dependents were determined as zero for healthy donors and 100 for CLL patients. The binary logistic regression model was carried out with a 95% confidence interval. Data was also analyzed using ROC curves of VDAC1 (B), SMAC/Diablo (D), Bcl-2 (F) and MAVS (H) expression levels in PBMCs samples from CLL patients and healthy donors. The AUC of the ROC curves for classifying CLL are presented in each curve.

A binary logistic regression model with a 95% confidence interval was employed to determine whether the expression level of each apoptosis-modulator protein could serve to assign test subjects to the healthy or CLL groups (Fig 6). In this analysis, we calculated sensitivity (predicted CLL patients/total CLL patients) and specificity (predicted healthy individuals/total healthy donors) values, based on VDAC1, SMAC/Diablo, Bcl-2 and MAVS (Fig 6), and for AIF, HK-I or Bax expression levels (S5 Fig). For VDAC1, Bcl-2, SMAC/Diablo and MAVS, the ability to predict the disease based on expression level of each protein was deemed to be over 90% (Fig 6, S2 Table). On the other hand, predicting disease based on the HK-I expression level was less supported (about 65%) (S5 Fig, S2 Table).

To analyze the performance of a binary classifier of each protein over-expressed in CLL patients, a ROC analysis was also performed (Fig 6). The AUC values of the ROC curves of 1.0, 0.996, 0.993 and 0.972 were obtained for SMAC/Diablo, Bcl-2, VDAC1 and MAVS, respectively, when CLL patients were compared with controls (Fig 6). AUC values for AIF and HK were 0.989, and 0.753, respectively (S5 Fig).

Such analysis clearly demonstrates the possibility of determining disease occurrence based on the expression levels of one or a combination of several of the apoptotic modulator proteins analyzed here, for CLL, confirming the potential of using the levels of these proteins as disease indicators with a small margin of error.

### Proteomics analysis allows distinction between patients in a stable disease state and those transferred to anti-cancer treatment or who died

**Fig 7:**
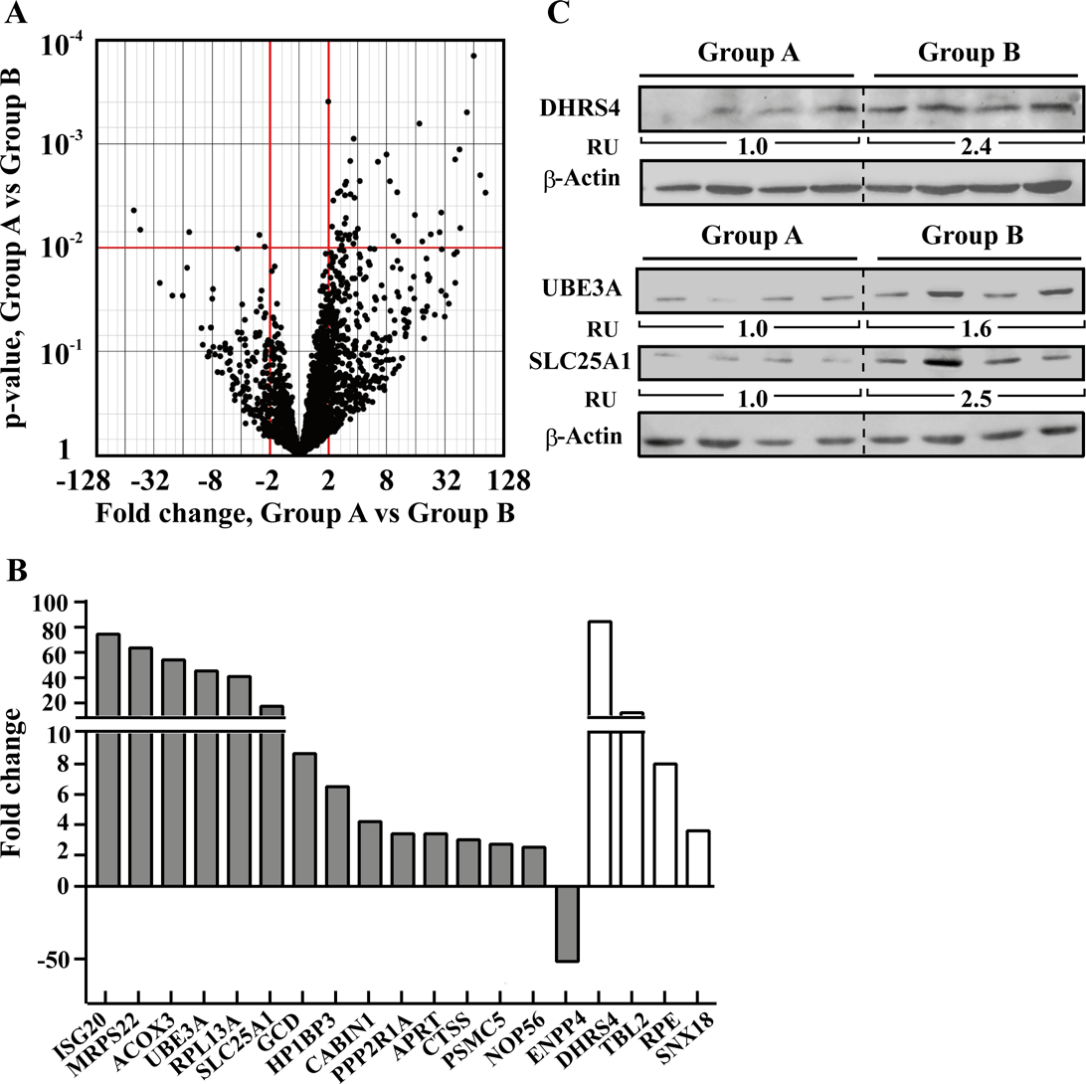
Differentially expressed proteins between CLL patients in a stable disease state and those transferred to anti-cancer treatments. **A.** A volcano plot shows proteins expression comparison between CLL patients in a stable disease state (group A) and patients transferred to anti-cancer treatments (group B). Presented are p-values and the magnitude of the difference in expression values (fold change) between groups B and A. Red lines indicate a nominal p-value cutoff of 0.01 and a fold change cutoff of |2|. Nineteen proteins passed the cutoff values of p-value < 0.01 and FC **≥**|2|. **B.** Differentially expressed proteins between groups A and B (p-value <0.01, FC **≥**|2|) are presented, divided as proteins reported to be associated (grey) or not associated (white) with cancer. **C.** Representative immunoblots shows the expression levels of DHRS4, UBE3A, and SLC25A1 in patients from groups A and B. Protein level was determined and normalized to β-Actin. Fold change is indicated for each group as relative units (RU).

Of the PBMCs derived from 19 CLL patients subjected to LC-HR-MS/MS analysis, 10 patients were in a stable disease state and needed no treatment 3 years after blood samples were obtained. Nine patients were subjected to treatment or died 0.5 to 36 months after collection of blood samples. Thus, to further identify proteins that allow to distinguish between patients in a stable disease state (group A) from those transferred to anti-cancer treatment or who died (group B), the protein expression profile of these two groups was analyzed. Hierarchical clustering of all 19 CLL samples could not properly separate between groups A and B (not shown). Yet, a volcano chart derived from a t-test with equal variances analysis revealed 50 proteins whose expression levels were significantly different between the two groups (FC ≥|2|, p-value <0.01) (Fig 7A).

Nineteen of the 50 proteins are presented with their fold change between groups A and B, their proposed function, subcellular localization and relevance to cancer (Table 2, Fig 7B). These include proteins that were previously associated with different types of cancer, of which mutation in one of them (ACOX3) was highly associated with a prediction of CLL outcome, while five proteins had no report connecting them to cancer.

**Table 2:** List of selected proteins differentially expressed between CLL patients in a stable disease state and those transferred to anti-cancer treatments, identified by LC-HR-MS/MS.

**Protein (UniProt accession)**	**Fold change/ P value**	**Proposed function and cellular localization**	**Relation to cancer**
ISG20- interferon stimulated exonuclease gene 20kDa (Q96AZ6)	74.6 2.0x10-3	Exhibit antiviral activity against RNA viruses (nucleus, cytoplasm)	Up-regulated in cervical cancer [70]
MRPS22—mitochondrial ribosomal protein S22 (P82650)	63.8 1.4x10^-4^	Protein synthesis (mitochondria)	Marker for epithelial breast cancer cells [71]
ACOX3- acyl-coenzyme A oxidase 3 (O15254)	54.2 5.0x10^-4^	Fatty acid beta-oxidation (peroxisome)	A SNP is highly associated with prediction of outcome of CLL [72], and possibly with prostate cancer [73]
UBE3A- ubiquitin protein ligase E3A (Q05086)	45.5 1.1x10^-3^	Targeting proteins for degradation (cytoplasm)	Over-expressed in breast cancer [74]. Catalyzes the ubiquitination of p53 [75]
RPl13a- 60S ribosomal protein L13a (P40429)	41.0 1.4x10^-3^	Associated with ribosomes but not with the canonical ribosome function, having extra-ribosomal functions (cytoplasm)	Involved in cancer stem cells [76]
SLC25A1- solute carrier family 25, member 1 (P53007)	17.5 6.4x10^-4^	Citrate transporter (mitochondria)	High expression in several tumor types [77]
GCD- glutaryl-Coenzyme A dehydrogenase (Q92947)	8.7 2.3x10^-3^	Involved in the degradation of L-lysine, L-hydroxylysine, and L-tryptophan (mitochondria)	Overexpressed in colon cancer [78]
HP1BP3- heterochromatin protein 1, binding protein 3 (Q5SSJ5)	6.5 1.5x10^-3^	Maintains heterochromatin integrity during G1/S progression (nucleus)	Indications for role in hypoxia-induced oncogenesis [79]
CABIN1- calcineurin binding protein1 (Q9Y6J0)	4.2 2.3x10^-3^	Inhibits calcineurin-mediated signal transduction (nucleus)	Down-regulated in nasopharyngeal carcinoma sensitizes them to genotoxic drugs. In B-cell lymphoma, regulates the oncogene CBL6 [80]
PPP2R1A- Protein Phosphatase 2, Regulatory Subunit A, Alpha (P30153)	3.4 1.5x10^-3^	Regulation of cell adhesion; second-messenger-mediated signaling; mitotic nuclear envelope reassembly and more (cytoplasm)	Marker for risk of breast cancer [81]
Aprt- adenine phosphoribosyl- transferase (P07741)	3.4 3.1x10^-3^	Involved in purine salvage pathway resulting in the formation of AMP (cytoplasm)	Sensitizes cells to treatment with *E*. *coli* purine nucleoside phosphorylase [82]
CTSS- cathepsin S (P25774)	3.0 2.5x10^-3^	Protease in MHC-II- mediated antigen presentation (lysosome)	Overexpressed in glioblastoma [83] Prognosis and predictor marker of chemotherapy benefit in adjuvant colorectal cancer [84]
PSMC5- proteasome (prosome, macropain) 26S subunit, ATPase 5 (also known as S8; p45; SUG1; TBP10; TRIP1) (P62195)	2.7 2.9x10^-3^	ATP-dependent degradation of ubiquitinated proteins (cytoplasm)	Its gene amplified during progression of cutaneous malignant melanoma [85]
Nop56- Nuclear protein 56 (O00567)	2.5 3.0x10^-3^	Involved in the 60S ribosomal subunit biogenesis (nucleus)	Necessary for Myc-induced cell transformation [86]
Enpp4- ectonucleotide pyrop-hosphatase/phosphodiesterase 4 (Q9Y6X5)	-51.8 4.4x10^-3^	Hydrolyze phosphodiester bonds, act as a procoagulant (cell membrane)	High expression in the in vivo metastatic osteosarcoma cells [87]
DHRS4- dehydrogenase /reductase (SDR family) member 4 (Q9BTZ2)	84.5 3.0x10^-3^	Reduces all-trans-retinal and 9-cis retinal (peroxisome)	**No direct association reported**
TBL2- transducin (beta)-like 2 (Q9Y4P3)	10.3 2.9x10^-3^	Associate with triglyceride metabolism (ER)	**No direct association reported**
RPE- ribulose-5-phosphate-3-epimerase (Q96AT9)	8.0 1.3x10^-3^	Pentose phosphate pathway (cytoplasm)	**No direct association reported**
Snx18- sorting nexin 18 (Q96RF0)	3.6 8.9x10^-4^	Endocytosis and vesicle trafficking during interphase and at the end of mitosis (cell membrane)	**No direct association reported**

Differentially expressed proteins between CLL patients in a stable disease state and those transferred to anti-cancer treatments (p-value <0.01, FC **≥|**2**|**) were filtered and proteins of interest are presented based on their relevance to cancer and potential as biomarkers. For each protein, its name, fold change and p-value are indicated, as are its function, subcellular localization and relevance to cancer.

To confirm this analysis and explore potential novel biomarkers for CLL progression, we examined the expression level of DHRS4, SLC25A1, and UBE3A in PBMCs from groups A and B by immunoblotting using specific antibodies (Fig 7C). The immunoblotting results confirmed the LC-HR-MS/MS data, with DHRS4, SLC25A1 and UBE3A expression increasing in both analyses.

## Discussion

As cancer-associated changes are not only mutation–related but can also appear as increases or decreases in protein expression levels or be associated with protein post-translational modification, biomarkers other than mutations should be identified and explored as early markers of the disease, as indicators of the disease state, and as predictive and prognostic gauges of treatment effectiveness. By applying LC-HR-MS/MS and immunoblot analyses to PBMCs isolated from healthy donors and CLL patients, we identified expression changes of a number of proteins related to cellular functions and pathways associated with CLL, and with mitochondria, apoptosis and metabolism (Fig 1B, Tables 1 and 2). The identified altered proteins can serve as biomarkers for early CLL diagnosis, and as potential new targets for development of new treatments for CLL.

### Alterations in protein expression between healthy and CLL patients—potential CLL biomarkers and potential new targets for CLL therapy

Using LC-HR-MS/MS-based protein profile analysis, we identified a battery of proteins that can distinguish between healthy individuals and CLL patients. When considering only the 59 proteins whose expression was found to be altered in 2 different LC-HR-MS/MS experiments, we identified several proteins that were previously found to be mutated in CLL or proposed as CLL diagnosis and prognosis markers (Table 1, group A). A second group of proteins associated with metabolism and apoptosis, including VDAC1, VDAC2 and AIF, were reported for some cancers other than CLL (Table 1, group B).

Finally, we identified several proteins with altered expression in CLL patients that were not previously reported to be associated with any cancer including CLL (Table 1, group C).

When focusing on the apoptotic modulating proteins, the binary logistic regression model [33] employed here demonstrated that SMAC/Diablo, AIF, MAVS, VDAC1 or Bcl-2 are with high potential as diagnostic markers of >90% accuracy when using Bivariance analysis, and AUC values of 0.97 to 1.0 in the ROC curve analysis for CLL diagnosis (Fig 6).

The differentially expressed proteins in CLL presented here are consistent with and contribute to the malignant phenotype of the cancer cells. As a single biomarker not always sufficient for cancer diagnosis, the additional proteins explored here with altered expression level in CLL may be used for CLL early diagnosis, prediction of disease progress and assessment of future treatment effectiveness.

Moreover, some of these proteins may be potential new targets for developing CLL treatment. Indeed, down-regulation of the RNA helicase DDX46, involved in pre-mRNA processing, such as pre-mRNA splicing, rRNA biogenesis, and transcription [61], and of the mitochondrial protein AK3, involved in maintaining the homeostasis of cellular nucleotides [63], resulted in profound inhibition of cell growth (Fig 2). Interestingly, DDX46 was shown to be required for multi-lineage differentiation of hematopoietic stem cells in zebrafish [88].

Finally, high DDX46 expression levels are significantly associated with shorter survival of CLL patients (S1 Fig) and thus, DDX46 can serve a marker of prognosis in these patients.

The second protein whose down-regulation inhibited cell growth was AK3 located exclusively in the mitochondrial matrix and involved in adenine metabolism. AK3 was not previously connected to CLL or any cancer, apart from the finding that deletion of chromosome 9p frequently occurs in bladder tumors [89], with chromosome 9 carrying AK1, AK2 and AK3. The mechanism(s) by which down-regulation of DDX46 or AK3 leads to decreased cell growth is not clear but will be explored in future study.

### CLL patients express high levels of apoptosis-modulating proteins, except the pro-apoptotic protein Bax

Alterations that attenuate apoptosis pathways are selected for during tumor initiation and progression, with ‘apoptosis evasion’ being considered an essential hallmark of cancer [4]. Hence, we analyzed the levels of apoptosis-modulating proteins, including Bcl-2, SMAC/Diablo, AIF, MAVS, and VDAC1, and found that all are markedly over-expressed in CLL (Figs 3 and 4). The underlying molecular mechanisms of altered expression of these apoptosis modulators in CLL are not known and form the basis for further studies. Our results confirm the over-expression of the anti-apoptotic protein Bcl-2, leading to clonal B cell expansion [5,7], and the decreased expression of the pro-apoptotic protein Bax in CLL [10,11,66], due to its abnormally increased degradation by the ubiquitin-proteasomal pathway [10].

### Alterations in protein expression between CLL patients—potential disease progression biomarkers

Analysis of CLL LC-HR-MS/MS results with respect to disease state, meaning patients in a stable condition or patients with progressing disease that required treatment or who died of the disease, revealed additional proteins that allow distinguishing between these two groups. Of the 50 proteins, the selected 19 were either associated with cancer, mainly breast cancer, or were not previously connected to cancer (5 proteins) and found here for the first time to be over-expressed in CLL patient-derived PBMCs (Table 2, Fig 7B). These include DHRS4, a peroxisomal protein which reduces all-trans-retinal and 9-cis retinal, SLC25A1, a mitochondrial tricarboxylate transporter mediating citrate-H+/malate exchange, and UBE3A, an E3 ubiquitin-protein ligase (Fig 7B and 7C). Additional proteins of interest that were over-expressed in CLL patients that required treatment at later stage of the disease progression (group B), include SNX18, involved in endocytosis and vesicle trafficking during interphase and at the end of mitosis [90], APRT, which sensitizes cells to treatment with E. coli purine nucleoside phosphorylase [82], Acox3-acyl-coenzyme A oxidase 3 (ACOX3), with a SNP shown to be associated with prediction of outcome of CLL [72] being highly (54.2-fold) over-expressed in CLL patients group B, as confirmed by immunoblotting (Table 2). Interestingly, ectonucleotide pyrop-hosphatase/phosphodiesterase 4 (ENPP4), involved in hydrolyze phosphodiester bonds and acting as a pro-coagulant and highly expressed in the metastatic osteosarcoma cells [87], was highly reduced (51.8-fold) in CLL group B (Table 2, Fig 7B).

These findings suggest that we could predict which CLL patients were in a stable condition with no need for treatment and those who should be treated. This molecular-based prognostic assay could help in decisions regarding the start of treatment and may complement current methods for predicting disease outcome and improve patient care.

### VDAC1 over-expression in CLL, a double-edged sword

One of the hallmarks of cancer essential to tumor progression is the reprogramming of energy metabolism [4]. VDAC1 is accepted as a master gatekeeper, regulating the flux of metabolites and ions between the mitochondria and the cytoplasm and thus controlling both cell energy and metabolic homeostasis [14,16,17,18,19,20,21,22]. VDAC1 was found to be over-expressed in several types of cancers, such as lung, cervical, thyroide and ovarey cancer [46,91,92], yet this report is the first to demonstrate VDAC1 over-expression in CLL (Figs 3 and 4 and Table 1). Indeed, in some patients, VDAC1 levels were increased up to 14-fold (Fig 3). Moreover, tight correlation between VDAC1 levels and the amount of cancerous cells, represented by CD19/CD5 double positive cells, was observed (Fig 5). Similarly, VDAC1 levels were correlated with the expression levels of the apoptosis-associated protein tested (S4 Fig).

To date, no studies have explored the potential diagnostic value of VDAC1 in cancer, and particularly, in CLL. VDAC1 over-expression offers numerous advantages to cancer cells, given its multi-functionality [14,16,17,22]. These include being the main transporter through the OMM of ATP and other metabolites and ions [17,22], involved in cholesterol transport [93], and presenting anchoring sites for HK, allowing direct transport of mitochondrial ATP for glucose phosphorylation, thereby increasing the glycolytic rate, a characteristic of cancer cells (i.e., the Warburg effect) [4]. Finally, over-expressed VDAC1 offers binding sites for Bcl-2 and Bcl-xL, allowing these proteins to mediate their anti-apoptotic activities [18,23,24,94].

VDAC1 is also directly involved in apoptosis [17,22,27]. VDAC1 over-expression was shown to induce apoptosis in the absence of any apoptotic stimuli [16,27]. In cancer cells, over-expressed anti-apoptotic proteins interact with VDAC1, thereby preventing VDAC1 pro-apoptotic activity. However, when association of these proteins with VDAC1 is interrupted, such as by VDAC1-based peptides [95], over-expressed VDAC1 becomes pro-apoptotic. Thus, VDAC1 over-expression in CLL can function as double-edged sword, on the one hand providing metabolic advantages to cancer cells and binding sites for over-expressed anti-apoptotic proteins, yet on the other hand, by interfering with anti-apoptotic protein binding to VDAC1, over-expressed VDAC1 displays pro-apoptotic activity, encouraging cell death [14,16,17,18,19,20,22,23,24,26,27].

### MAVS over-expression in CLL patients as an anti-apoptotic defense strategy

MAVS not only plays a pivotal role in the induction of anti-viral and inflammatory pathways but is also involved in the coordination of apoptotic and metabolic functions [96]. MAVS is active only when bound to mitochondria, specifically to VDAC1 [97], and is proposed to regulate cell death by various mechanisms [28,98]. Here, we have demonstrated, for the first time that MAVS is over-expressed in CLL (Figs 3 and 4). This may suggest that MAVS over-expression offers another unidentified strategy by which CLL cells resist apoptosis, or fulfil another function with advantage to cancer cells. MAVS over-expression in CLL may point to MAVS as a diagnostic marker, as well as a new possible target for CLL therapy.

### The paradox of over-expression of the pro-apoptotic proteins SMAC/Diablo and AIF in CLL

As shown here, SMAC/Diablo is also over-expressed in CLL (Figs 3 and 4). Moreover, SMAC/Diablo levels were correlated with cancerous cell levels (CD19/CD5) (Fig 5). This is surprising since SMAC/Diablo is a pro-apoptotic protein released from mitochondria during apoptosis that counters the activities of inhibitor of apoptosis proteins (IAPs) by releasing caspases bound to such proteins [68]. It has been shown that SMAC/Diablo mRNA and protein expression levels are reduced in hepatocellular carcinomal cells, as opposed to normal hepatic tissue [99]. However, others found higher SMAC/Diablo expression in adenocarcinomas, as compared to squamous cell tumors, and in cervical cancer patients [100] and in gastric adenocarcinomas [101]. Finally in renal cell carcinoma, SMAC/Diablo mRNA levels in meningiomal cells were higher than in normal cells [102].

This discrepancy between increased SMAC/Diablo levels in CLL and its pro-apoptotic activity [68] may result from another, as yet unidentified, activity of SMAC/Diablo other than countering the activities of IAPs.

AIF is also over-expressed in CLL (Figs 3 and 4). AIF is a flavin adenine dinucleotide-containing, NADH-dependent oxidoreductase residing in the mitochondrial intermembrane space [67]. Upon apoptosis induction, AIF undergoes proteolysis, is released from the mitochondria and translocates to the nucleus, where it triggers chromatin condensation and large-scale DNA degradation [67]. As a pro-apoptotic protein, it is unclear why AIF is over-expressed in cancer cells. AIF has also emerged as a protein critical for development, as homozygous AIF knockout in mice is lethal [103]. The pro-survival activity of AIF was proposed to be related to oxidative phosphorylation, ROS detoxification, redox-sensing, mitochondrial morphology and cell cycle regulation [67,104]. AIF over-expression in CLL may offer an advantage to cancer cells via these processes. Both SMAC/DIBLO and AIF over-expressed in CLL can serve as CLL biomarkers and as possible new targets for CLL therapy.

## Conclusions

The expression levels of many proteins identified by LC-HR-MS/MS analysis are highly altered in CLL patients, reflecting cancer cells reprogramming including of metabolic and anti-apoptotic features. Some of these proteins were previously identified as associated with CLL or other cancers, but some are newly identified as related to CLL. Down-regulation of two of the newly identified proteins resulted in cell growth inhibition. Based on the levels of some of these proteins in CLL patients, it is possible to distinguish between patients in a stable disease state and those eventually subjected to anti-cancer treatments or who had died of the disease. Furthermore, immunoblotting analysis further exposed several proteins (e.g., VDAC1, MAVS, AIF and SMAC/Diablo) as highly over-expressed in CLL and pointed to new functions for these pro-apoptotic proteins. The use of these advanced molecular diagnostics protein-based biomarkers may facilitate accurate diagnosis, prognostic prediction and monitoring response to treatment and could contribute to individualized CLL treatment. Finally, these newly identified biomarkers may also serve as targets for the development of new treatments for CLL.

## Supporting Information

S1 Fig Prognostic value of AP3B, ADH5, DDX46 and AK3 levels in CLL patients. Kaplan-Meier survival curves for CLL patients are presented for patients with high expression (red line) and low expression (blue line) of AP3B (A), ADH5 (B), DDX46 (C) and AK3 (D). Tick marks represent the survival time for patients that did not participate in the full duration of the experiment. Survival time was measured from date of diagnosis to date of death for patients who died and to the date of the last follow-up for those who were alive at the time of the analysis. A difference between two curves was considered statistically significant when P < 0.05.
(TIF)

S2 Fig Immunocytochemical staining of VDAC1, SMAC/Diablo and MAVS, PBMCs. For immunocytochemical staining of VDAC1, SMAC/Diablo and MAVS, PBMCs (1×10^6^) derived from healthy donors (**A**) and CLL patients (**B**) were PBS–washed, transferred to coverslips, fixed with paraformaldehyde (4%, 15 min), immuno-stained using anti-VDAC1 (**a,d**), anti-SMAC/Diablo (**b,e**) or anti-MAVS (**c,f**) antibodies, followed by incubation with Cy2-conjugated secondary antibodies and visualization by confocal microscopy (bar = 10 μm). Arrows point to high intensity fluorescence surrounding the nucleus and occupying the cytoplasm.
(TIF)

S3 Fig Similar levels of mitochondria in PBMCs derived from CLL patients and healthy donors. The amounts of mitochondria in PBMCs derived from CLL patients and healthy donors were analyzed using MitoTracker green (unstained (**a, b),** stained (**c, d**)). Results are representative of three similar experiments.
(TIF)

S4 Fig VDAC1 expression level is correlated with the level of apoptosis-related proteins. Correlation between the relative expression of VDAC1 and the apoptosis-related proteins SMAC/Diablo (**A,** n = 21) and Bcl-2 (**B,** n = 28) in CLL patients was determined by linear regression, with the data points fitting the line with the indicated R^2^. All analyses were performed with 95% confidence. VDAC1, SMAC/Daiblo, Bcl-2 levels were assayed as described in the legend to Fig 3.
(TIF)

S5 Fig Binary logistic regression testing for specificity, sensitivity and overall CLL predication based on the relative expression of apoptosis-related proteins. Bivariance analysis was performed based on the relative expression of apoptosis-related proteins, considered as independent variables. Relative protein expression levels were those presented in Fig 2, with data from healthy donor (●) and CLL patient (O) are represented for AIF (A), HK-I (B) and BAX (C). The dependents were determined as zero for healthy donors and 100 for CLL patients. The binary logistic regression model was carried out with a 95% confidence interval. ROC curves of AIF (D), HK-I (E) and Bax (F) expression levels in PBMCs samples from CLL patients and healthy donors. The AUC of the ROC curves for classifying CLL are presented in each curve.
(TIF)

S1 Table Clinical characteristics of patients with B-CLL. All patients were untreated at the time of this study. Patients’ average age was 69 years, composed 14 males and 17 females. The T cell specific zeta-associated protein 70 (Zap 70) is an intracellular tyrosine kinase. ZAP-70 is the gene used to distinguish the CLL subtypes. The expression of ZAP-70 and the co-expression of the T-cell antigen CD5 and B-cell surface antigens CD19 were analyzed in peripheral-blood samples from the patients with CLL using specific antibodies and flow cytometry. Positive (over 15%) and negative (less than 14%) signals are indicated by + and–, and ND indicates not determined. About 13% of the tested samples were ZAP-positive.
(TIF)

S2 Table Binary logistic regression testing for specificity, sensitivity and overall CLL predication based on the relative expression of apoptosis-related proteins. For details see legend to Fig 6.
(TIF)

## Abbreviations

AIF: apoptosis inducing factor
CLL: chronic lymphocytic leukemia
HK: hexokinase
LC-HR-MS/MS: liquid chromatography high-resolution mass spectrometry
MAVS: mitochondrial anti-viral signaling protein
OMM: outer mitochondria membrane
PBMCs: peripheral blood mononuclear cells
SMAC/Diablo: second mitochondria-derived activator of caspases/ Direct IAP-binding protein
VDAC1: voltage-dependent anion channel 1
