# Supplementary figures and images for: Novel Biomarker Proteins in Chronic Lymphocytic Leukemia: Impact on Diagnosis, Prognosis and Treatment
Source: PLoS One. 2016 Apr 14;11(4):e0148500. doi: 10.1371/journal.pone.0148500 (PMC4831809; doi:10.1371/journal.pone.0148500)

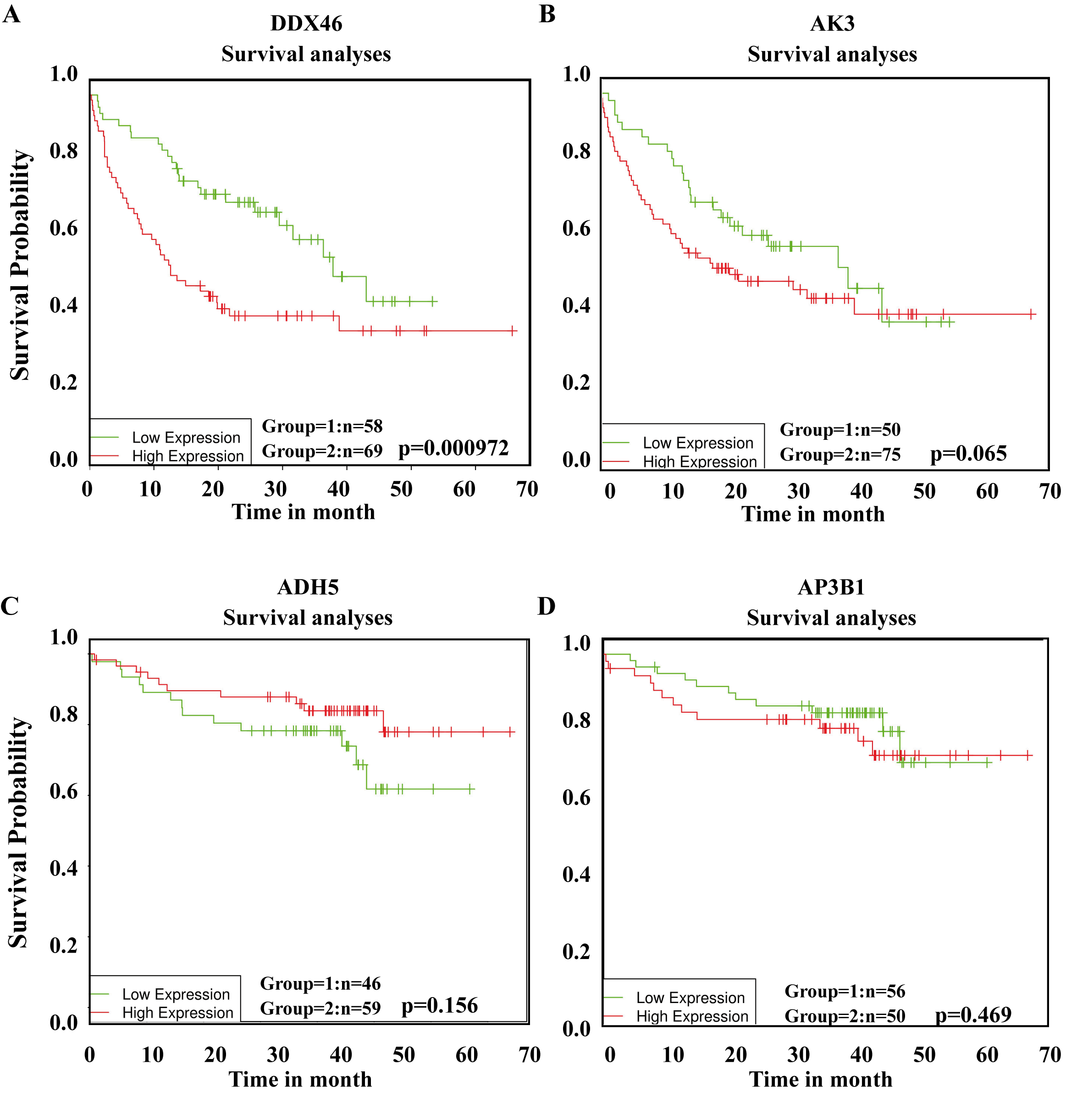

Supplement: S1 Fig — Kaplan-Meier survival curves for CLL patients are presented for patients with high expression (red line) and low expression (blue line) of AP3B (A), ADH5 (B), DDX46 (C) and AK3 (D). Tick marks represent the survival time for patients that did not participate in the full duration of the experiment. Survival time was measured from date of diagnosis to date of death for patients who died and to the date of the last follow-up for those who were alive at the time of the analysis. A difference between two curves was considered statistically significant when P < 0.05. (TIF) [file pone.0148500.s001.tif]

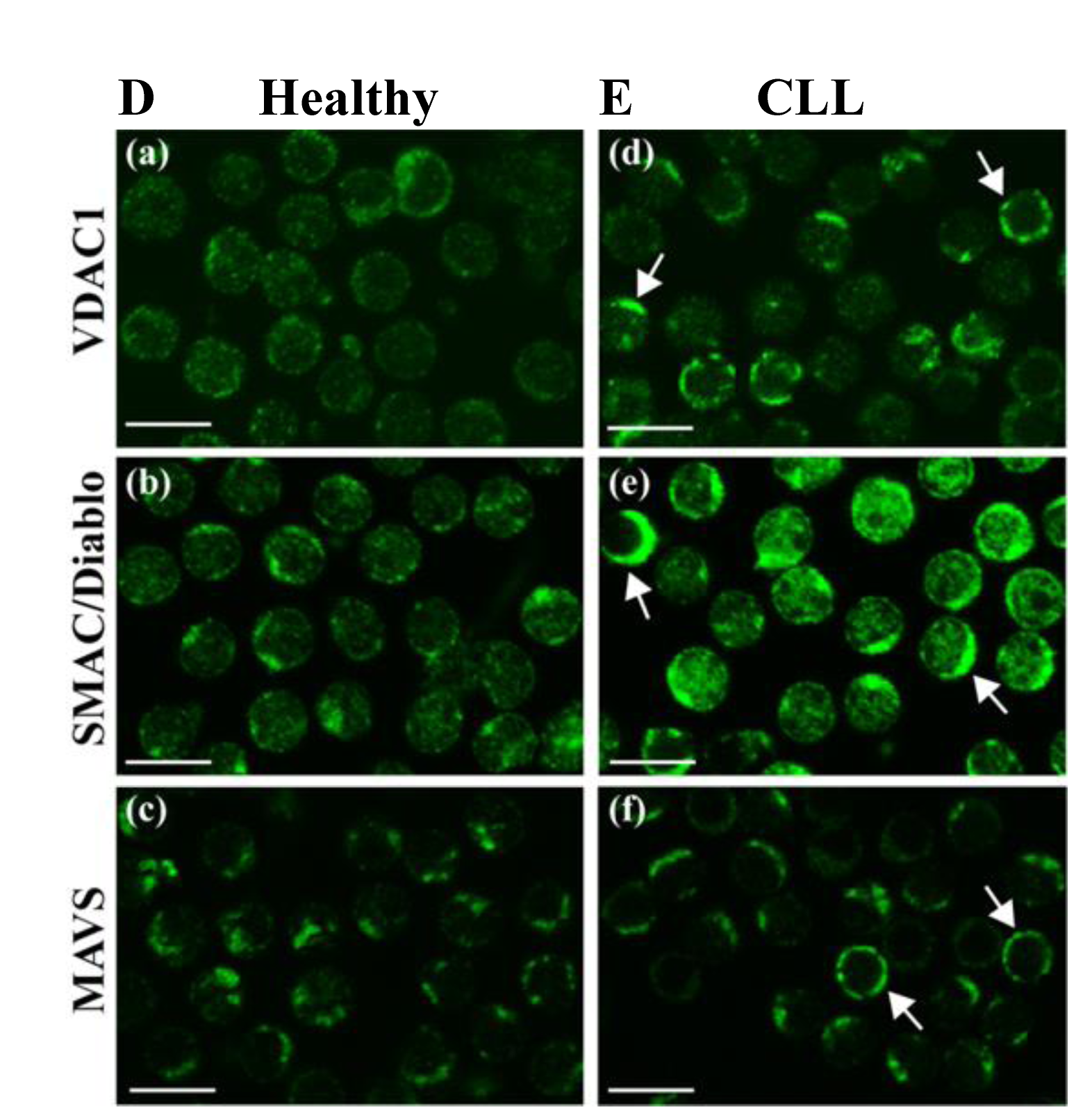

Supplement: S2 Fig — For immunocytochemical staining of VDAC1, SMAC/Diablo and MAVS, PBMCs (1×10^6^) derived from healthy donors (A) and CLL patients (B) were PBS–washed, transferred to coverslips, fixed with paraformaldehyde (4%, 15 min), immuno-stained using anti-VDAC1 (a,d), anti-SMAC/Diablo (b,e) or anti-MAVS (c,f) antibodies, followed by incubation with Cy2-conjugated secondary antibodies and visualization by confocal microscopy (bar = 10 μm). Arrows point to high intensity fluorescence surrounding the nucleus and occupying the cytoplasm. (TIF) [file pone.0148500.s002.tif]

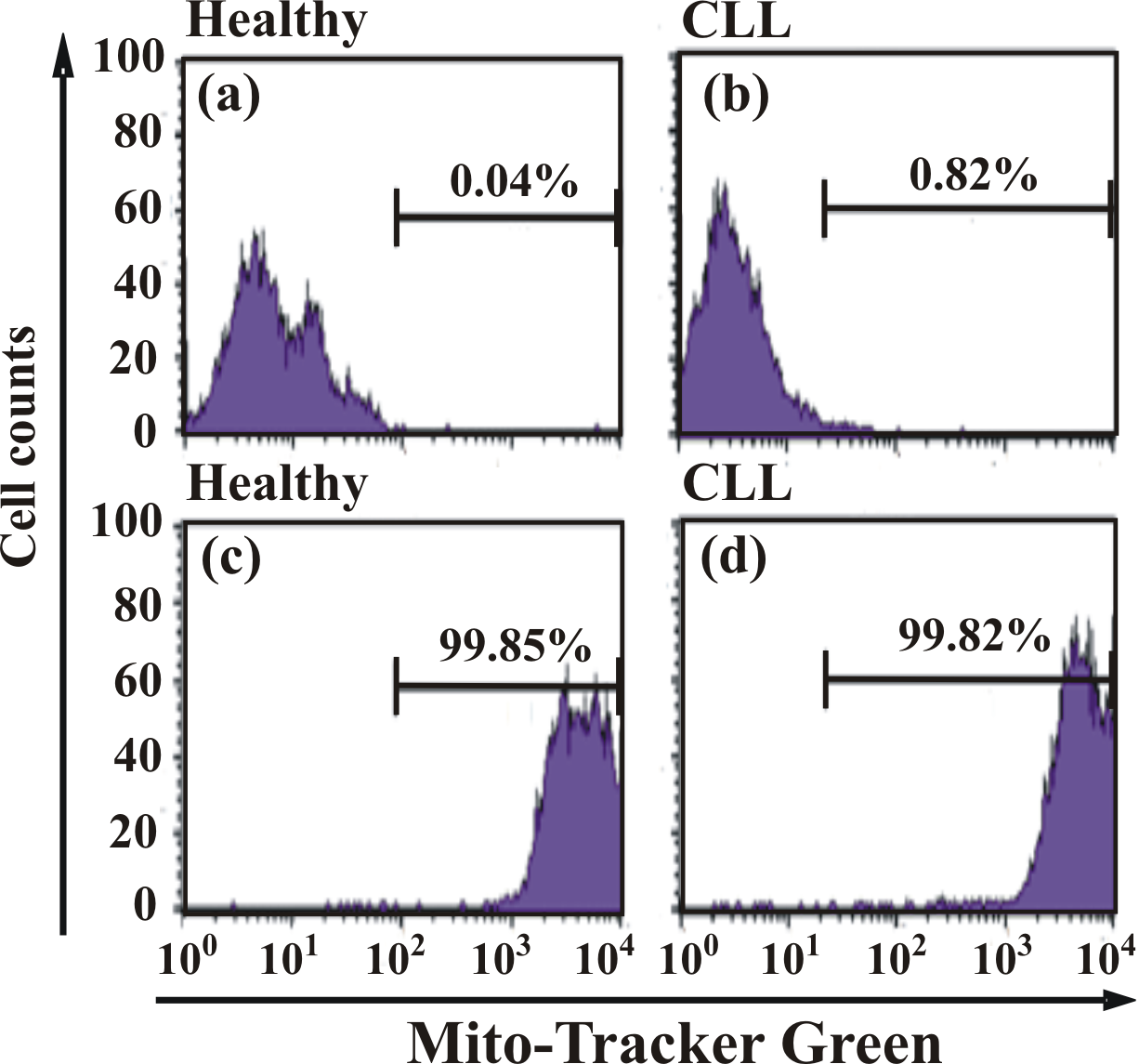

Supplement: S3 Fig — The amounts of mitochondria in PBMCs derived from CLL patients and healthy donors were analyzed using MitoTracker green (unstained (a, b), stained (c, d)). Results are representative of three similar experiments. (TIF) [file pone.0148500.s003.tif]

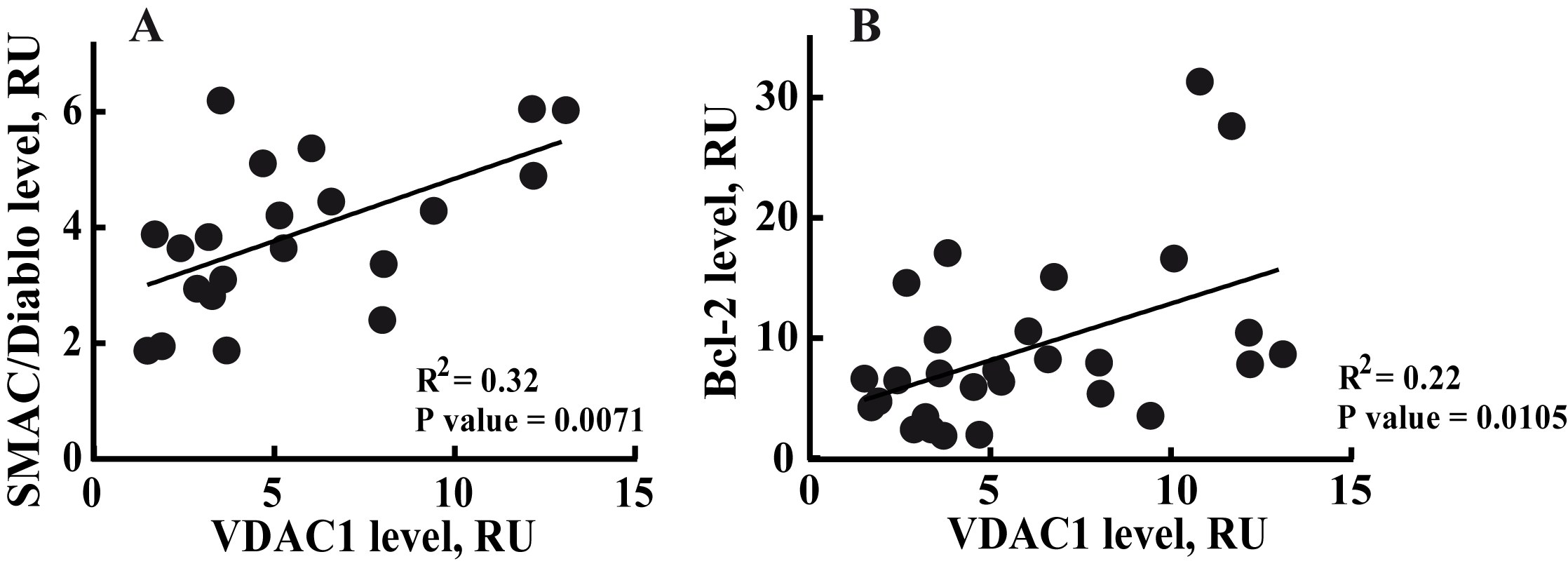

Supplement: S4 Fig — Correlation between the relative expression of VDAC1 and the apoptosis-related proteins SMAC/Diablo (A, n = 21) and Bcl-2 (B, n = 28) in CLL patients was determined by linear regression, with the data points fitting the line with the indicated R2. All analyses were performed with 95% confidence. VDAC1, SMAC/Daiblo, Bcl-2 levels were assayed as described in the legend to Fig 3. (TIF) [file pone.0148500.s004.tif]

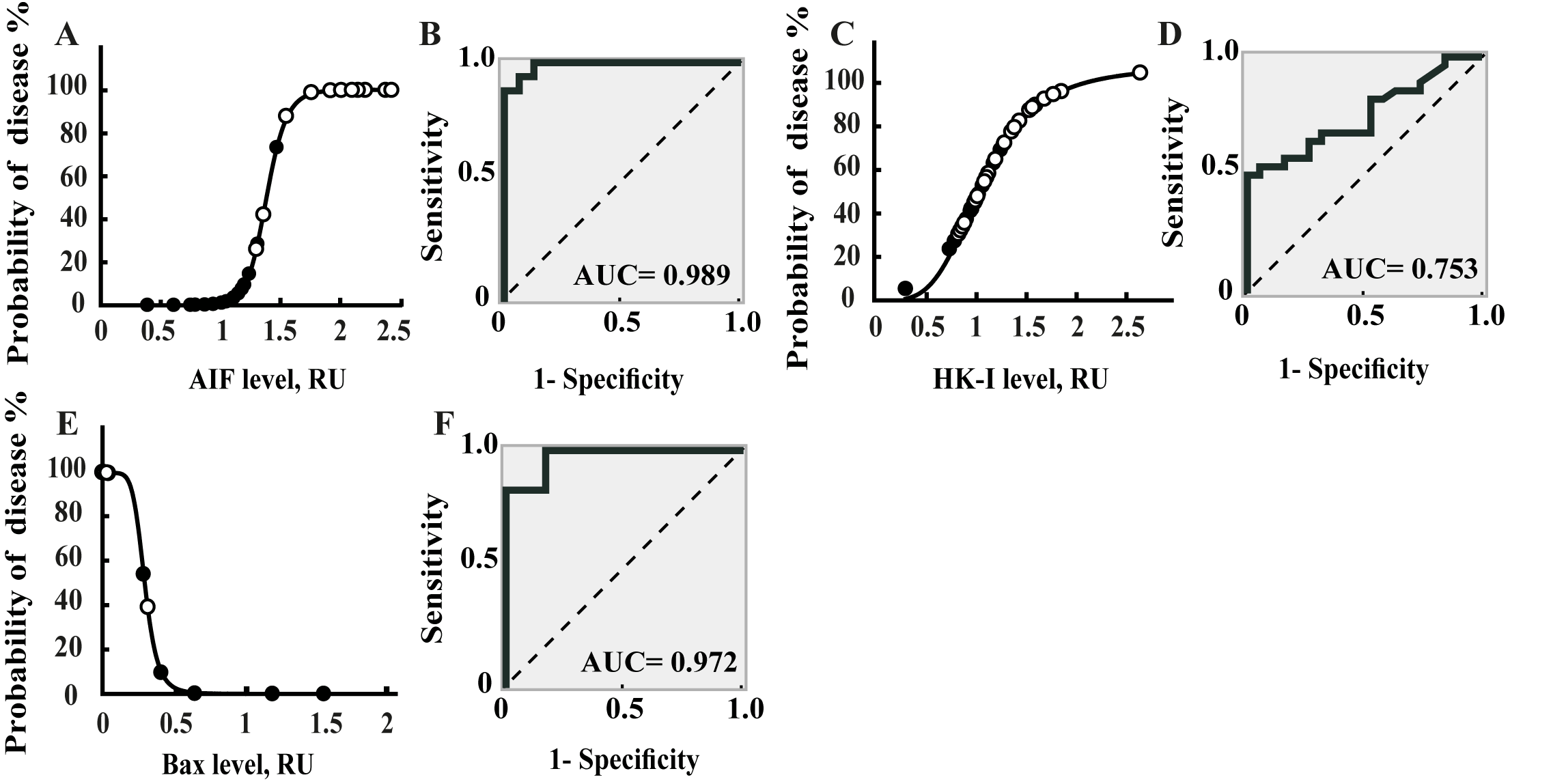

Supplement: S5 Fig — Bivariance analysis was performed based on the relative expression of apoptosis-related proteins, considered as independent variables. Relative protein expression levels were those presented in Fig 2, with data from healthy donor (●) and CLL patient (O) are represented for AIF (A), HK-I (B) and BAX (C). The dependents were determined as zero for healthy donors and 100 for CLL patients. The binary logistic regression model was carried out with a 95% confidence interval. ROC curves of AIF (D), HK-I (E) and Bax (F) expression levels in PBMCs samples from CLL patients and healthy donors. The AUC of the ROC curves for classifying CLL are presented in each curve. (TIF) [file pone.0148500.s005.tif]

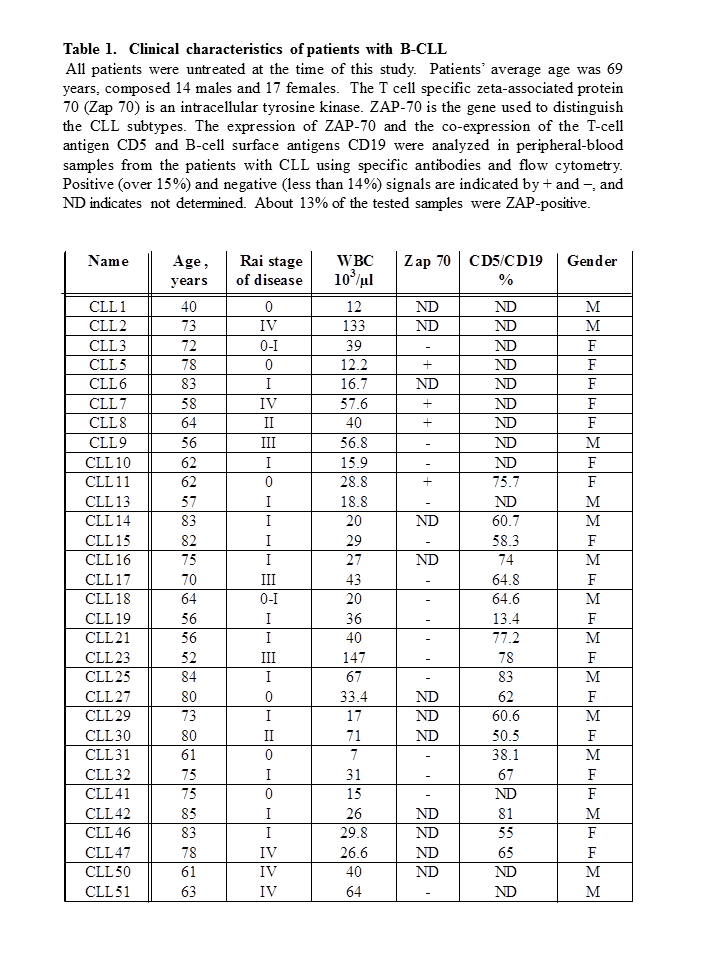

Supplement: S1 Table — All patients were untreated at the time of this study. Patients’ average age was 69 years, composed 14 males and 17 females. The T cell specific zeta-associated protein 70 (Zap 70) is an intracellular tyrosine kinase. ZAP-70 is the gene used to distinguish the CLL subtypes. The expression of ZAP-70 and the co-expression of the T-cell antigen CD5 and B-cell surface antigens CD19 were analyzed in peripheral-blood samples from the patients with CLL using specific antibodies and flow cytometry. Positive (over 15%) and negative (less than 14%) signals are indicated by + and–, and ND indicates not determined. About 13% of the tested samples were ZAP-positive. (TIF) [file pone.0148500.s006.tif]

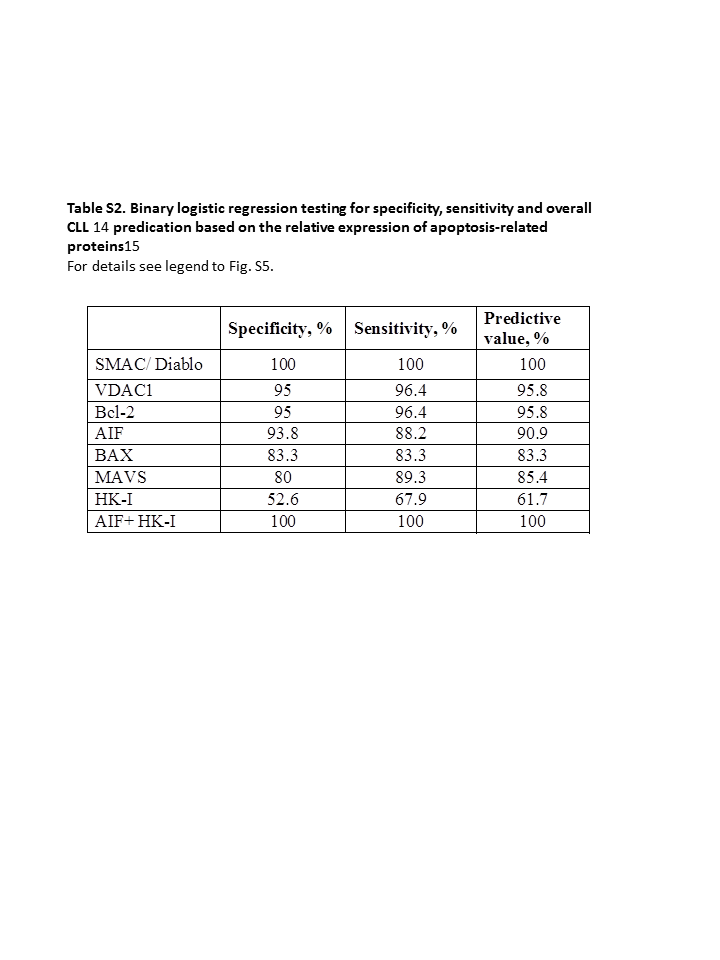

Supplement: S2 Table — For details see legend to Fig 6. (TIF) [file pone.0148500.s007.tif]
